# The 8th International Conference on Biomedical Engineering and Pharmaceutical Sciences (ICBEPS2023)

**DOI:** 10.1097/MD.0000000000035708

**Published:** 2023-12-29

## BEPS23001: PROGNOSTIC RISK FACTORS OF T2, T3, AND T4 THORACIC ESOPHAGEAL CANCER AND THEIR RELATIONSHIP WITH IMPROVED SURVIVAL AFTER RADIOTHERAPY: A PROPENSITY-SCORE-MATCHED LONGITUDINAL COHORT STUDY

Linghua Wei^a,b^, Fengti Liu^c^, Zhengsheng Yin^a^, Wei Zhao^d^, Yaxi Duan^a^, Xiang Li^a^, Xiaomin Wu^e^, Chengzhen Xu^a,b^

^*a*^ *College of Computer Science and Technology, Huaibei Normal University, 100 Dongshan Road, Xiangshan District, Huaibei, Anhui 235000, China,* ^*b*^ *Anhui Engineering Research Center for Intelligent Computing and Application on Cognitive Behavior, Huaibei, Anhui 235000, China,* ^*c*^ *Equipmemt Department, People’s Hospital of Rizhao, 126 Tai’an Road, Donggang District, Rizhao, Shandong 276826, China,* ^*d*^ *Imaging Department, Huaibei People’s Hospital, No.66 Huaihai Road, Huaibei, Anhui 235000, China,* ^*e*^ *College of Life Sciences, Huaibei Normal University, 100 Dongshan Road, Xiangshan District, Huaibei, Anhui 235000, China.*

**Background:** Esophageal cancer is a common malignant tumor of the digestive tract. In the past 40 years, the 5-year survival rate has slightly improved, but the overall survival rate is still relatively low. The effectiveness of esophageal cancer surgery is related to many factors, including the patient’s age, stage, and surgical method (including neoadjuvant chemotherapy and neoadjuvant chemoradiotherapy). This study aimed to investigate the prognostic risk factors of T2-T4 thoracic esophageal cancer and the survival benefit of common treatment modalities to provide a basis for selecting the most appropriate treatment.

**Subjects and Methods:** This population-based retrospective cohort study collected data of patients diagnosed with T2-T4 thoracic esophageal cancer between 2010 and 2016 in the Surveillance, Epidemiology, and End Results database. The SEER * Stat 8.3.8 software was used for collecting data. Patients were divided according to those who had surgery and those who had radiotherapy. Survival curves were generated using the Kaplan–Meier (KM) method and compared using the log-rank test. Cox regression analysis was used to analyze the independent risk factors for prognosis. The effects of common treatment methods were analyzed according to their survival benefit.

**Results:** A total of 4998 patients were included; of them, 1953 and 3045 patients were in the surgical and non-surgical groups, respectively, and 3951 and 1047 patients were in the radiotherapy and non-radiotherapy groups, respectively. There was no significant difference in survival between the surgical and non-surgical groups (P=0.79). There was no significant difference in survival between those with T2 (P=0.47) and T4 (P=0.66) disease between the radiotherapy and non-radiotherapy groups, but there was between the radiotherapy and non-radiotherapy groups in patients with T3 disease (P=0.0019). Radiotherapy is an independent risk factor for the prognosis of T3 esophageal cancer, but not for T2 and T4 esophageal cancer.

**Conclusions:** Surgery has no effect on survival in patients with T2-T4 thoracic esophageal cancer. Radiotherapy and T3 disease are independent prognostic risk factors for patients with T2-T4 thoracic esophageal cancer. These results provide a reliable basis for clinical treatment of patients with T2 -T4 thoracic esophageal cancer.

**Acknowledgments**

This work was supported by the Natural Science Foundation of Anhui Province (1908085QF286, 1808085QF181), the Natural Science Foundation of Anhui Provincial Department of Education (KJ2020B15, KJ2020A0031).

## BEPS23002: COMPREHENSIVE ANALYSIS OF SINGLE-CELL RNA SEQUENCING DATA OF HUMAN BLADDER TISSUE SAMPLES IN NORMAL AND CANCER STATES, WITH AND WITHOUT ANTI-PD-1 TREATMENT

Zhuo Wang^a,b^, Yuxuan Pang^a,c^, Ziji Liang^d^, Haodong Chen^d^, Jiexin Pan^d^, Meng Zhang^b^, Tzong-Yi Lee^e,f^, Hsien-da Huang^a,b^, Xingzhi Li^d^

^*a*^ *Warshel Institute for Computational Biology, School of Medicine, The Chinese University of Hong Kong, Shenzhen, Shenzhen, Guangdong, 518172, China,* ^*b*^ *School of Medicine, The Chinese University of Hong Kong, Shenzhen, Shenzhen, Guangdong, 518172, China,* ^*c*^ *School of Science and Engineering, The Chinese University of Hong Kong, Shenzhen, Shenzhen, Guangdong, 518172, China,* ^*d*^ *Department of Urology, Longgang District People’s Hospital of Shenzhen, Shenzhen, Guangdong, 518172, China,* ^*e*^ *Institute of Bioinformatics and Systems Biology, National Yang Ming Chiao Tung University, Hsinchu, 300093, Taiwan,* ^*f*^ *Center for Intelligent Drug Systems and Smart Bio-devices (IDS2B), National Yang Ming Chiao Tung University, Hsinchu, 300093, Taiwan.*

**Background:** Single-cell RNA sequencing (scRNA-seq) has emerged as a powerful technology to study cellular heterogeneity in complex tissues. Bladder cancer is a prevalent and challenging disease, and understanding the cellular response to anti-PD-1 treatment in the bladder tumor microenvironment is crucial for developing effective therapies. In this study, we aimed to investigate the effects of anti-PD-1 treatment on cellular responses in human bladder tissue samples obtained from both normal and cancer states.

**Subjects and Methods:** To comprehensively analyze scRNA-seq data, we obtained human bladder tissue samples from individuals in different conditions: normal, cancerous, with and without anti-PD-1 treatment. We employed state-of-the-art bioinformatics techniques to identify specific cell clusters affected by anti-PD-1 treatment and explore changes in gene expression signatures and cell-cell communication.

**Results:** Our analysis revealed a specific epithelial cell cluster that exhibited significant changes in response to anti-PD-1 treatment. This finding provided valuable insights into the underlying mechanisms and potential therapeutic targets of anti-PD-1 treatment in bladder cancer. Notably, we observed a higher cytolytic activity (CYT) score, determined by increased GZMA and PRF1 mRNA expression levels, in anti-PD-1-treated T cells, indicating an enhanced immune response against cancer cells. Additionally, cell-cell communication analysis unveiled that anti-PD-1 treatment profoundly increased the number and strength of interactions among fibroblasts, endothelial cells, and other cell types in the bladder tissue. These findings suggest that modulating these interactions may contribute to improved therapeutic outcomes in bladder cancer.

**Conclusions:** Our study provides a comprehensive view of the transcriptional landscape of human bladder tissue in both normal and cancer states, as well as the effect of anti-PD-1 treatment on the bladder tumor microenvironment. The observed alterations in gene expression signatures, enrichment of specific immune cell subsets, and enhanced cell-cell communication highlight the potential immunomodulatory effects of anti-PD-1 treatment. These insights offer valuable opportunities for the development of targeted therapies and personalized medicine for bladder cancer patients. Further investigation into the junctional adhesion molecule (JAM) signaling pathway and its role in anti-PD-1 treatment may shed more light on potential therapeutic mechanisms. Overall, our findings contribute to improving treatment strategies and advancing our understanding of bladder cancer biology.

**Acknowledgments**

This work was supported by Longgang District Health Science and technology project (LGKCYLWS2021000011). The work was also supported by the Warshel Institute for Computational Biology, School of Medicine, The Chinese University of Hong Kong, Shenzhen, China; Shenzhen-Hong Kong Cooperation Zone for Technology and Innovation (HZQB-KCZYB-2020056,P2-2022-HDH-001-A); National Natural Science Foundation of China (No. 32070674); Guangdong Young Scholar Development Fund of Shenzhen Ganghong Group Co., Ltd. (2021E0005, 2022E0035); Key Program of Guangdong Basic and Applied Basic Research Fund (Guangdong–Shenzhen Joint Fund) [2020B1515120069]. Natural Science Foundation of Guangdong (2023A1515011861).

## BEPS23003: COMPARISON OF TWO METHODS FOR PREPARING SERUM BY REMOVING FIBRINOGEN

Wei Wu^a^, Yi Liu^b^, Yehuan Zheng^c^, Yuxuan Jin^b^, Changzhu Hu^d^

^*a*^ *Department of Blood Bank, The Second Affiliated Hospital of Zhengzhou University, Zhengzhou, 451191, China,* ^*b*^ *Department of Optometry, School of Medical Technology and Engineering, Zhengzhou Railway Polytechnic, Zhengzhou, 451191, China,* ^*c*^ *Medical Department of Xi’an Jiaotong University, Xi’an, 710049, China,* ^*d*^ *Zhang Jiang Institute of Science and Technology, Fudan University, Pudong, Shanghai, 200120, China.*

**Background:** Fibrinogen is an important coagulation factor, which plays a key role in the process of blood coagulation. During the coagulation process, Ca2+ and thrombin are both important. However, the difference between the calcium restoration method and the thrombin method is unknow.

**Subjects and Methods:** In order to prepare high-quality and stable biochemical substrates, this article explores the use of different concentrations of calcium chloride and thrombin to prepare serum from plasma. The recalcification method and the addition of thrombin were used to remove fibrinogen from plasma, and serum was prepared for biochemical substrates. The serum substrates prepared by the two methods were compared in Differences in performance indicators such as quality and stability.

**Results:** The results showed that using sodium citrate as an anticoagulant in plasma, adding calcium chloride 20mmol/L, standing overnight at 4°C or adding thrombin 20 IU/mL, 37°C for 2h to remove fibrinogen, the serum was treated, the fibrinogen test result was less than 0.05g/L, and no fibrin was precipitated after repeated freezing and thawing several times. The concentration of calcium chloride is greater than 20mmol/L, and standing at 4 °C can effectively remove fibrinogen (<0.05g/L) in plasma, and freeze-thaw 5 times, and there will be no fibrin precipitation. The concentration of thrombin is greater than 20IU/mL, and standing in a 37°C water bath for 2h can effectively remove fibrinogen (<0.05g/L) in plasma, and freeze-thaw 5 times, without fibrin precipitation. The serum prepared by the two fibrinogen removal methods was light yellow to yellow clear liquid, and the clinical biochemical indexes including ions, enzymes, lipids and other differences were not significant. Even if stored at 4 °C for 7 days, or -20 °C for 180 days, or even frozen and thawed 5 times, the difference in biochemical indicators was not significant.

**Conclusions:** There is no difference in the clinical biochemical testing of serum prepared by the two methods, and both have good stability and meet clinical needs. Although the calcium chloride method has low cost, it still requires dialysis and other treatments to remove calcium ions from the serum. The thrombin method can meet the demand by removing fibrin and concentrating it appropriately. However, this method has a high cost and is not suitable for mass production.

## BEPS23004: ANALYSIS OF THE THERAPEUTIC EFFICACY OF COMBINED VAGINAL MUCOSAL PRESERVATION PERINEOPLASTY AND CARBON DIOXIDE FRACTIONAL LASER TREATMENT FOR POSTPARTUM VAGINAL LAXITY

Yuxiang Ke^a^, Jinlan He^a^

^*a*^ *Department of Gynaecology and Obstetrics, Yiling People’s Hospital of Yichang, Yichang 443100, China.*

**Background:** Vaginal laxity is a pelvic floor disorder commonly observed in women who have undergone vaginal childbirth. It can lead to various complications and have a detrimental impact on women’s physical and mental health. There are various treatment methods available for vaginal laxity, and their effectiveness may vary. Investigating the treatment methods and their efficacy for vaginal laxity is not only beneficial for improving patients’ pelvic floor muscle function but also plays a positive role in improving sexual function and addressing psychological issues caused by vaginal laxity.

**Subjects and Methods:** This study aims to analyze the therapeutic efficacy of combined vaginal mucosal preservation perineoplasty and carbon dioxide fractional laser treatment for postpartum vaginal laxity. The study included 20 patients with vaginal laxity who received treatment at XX Hospital from January 2020 to December 2022. Vaginal mucosal preservation perineoplasty and carbon dioxide fractional laser treatment were performed sequentially (3 months after perineoplasty). Vaginal laxity was assessed using the Vaginal Laxity Questionnaire (VLQ), and female sexual function was evaluated using the Female Sexual Function Index (FSFI) before surgery, after surgery, and 3 months after carbon dioxide fractional laser treatment. The clinical effects of combined vaginal mucosal preservation perineoplasty and carbon dioxide fractional laser treatment for vaginal laxity were analyzed.

**Results:** A total of 20 patients with vaginal laxity were included in this study, all of whom had varying degrees of vaginal laxity (vaginal width greater than or equal to 3 fingers). After vaginal mucosal preservation perineoplasty, vaginal laxity and female sexual function, including sexual desire, sexual arousal, vaginal lubrication, orgasm, and sexual satisfaction, significantly improved (P<0.05). After 3 months of combined carbon dioxide fractional laser treatment, there was significant improvement (P<0.05) in vaginal laxity, sexual desire, sexual arousal, vaginal lubrication, orgasm, and sexual satisfaction compared to undergoing vaginal tightening surgery alone, except for improvement in dyspareunia (P=0.145).

**Conclusions:** Combined vaginal mucosal preservation perineoplasty and carbon dioxide fractional laser treatment yield satisfactory results in improving symptoms of vaginal laxity in women with few complications. These findings are worthy of clinical promotion.

## BEPS23005: HEALTH MANAGEMENT ACTIVITIES, ELDERLY CARE MODE PREFERENCES AND MENTAL HEALTH PROMOTION AMONG THE ELDERLY WITH CHRONIC DISEASES IN JIANGSU PROVINCE

Huiyong Song^a^, Xiangjun Li^a^

^*a*^ *Nanjing University of Chinese Medicine, Jiangsu 210023, China.*

**Background:** To explore the classification of health management activities and elderly care modes and to analyze the associations between health management activities and preferences for elderly care modes and the impact of diversified elderly care mode on the mental health of the elderly.

**Subjects and Methods:** Data were obtained from the Sixth National Health Service Survey, and a chi-square test and multi-logistic regression model were used to analyze the relationship between health management activities and preferences for the various modes of elderly care.

**Results:** Elderly people who lack health records, have been assigned a family doctor and have received many follow-up service items have a higher likelihood to receive home-based elderly care; elderly people with three or more chronic diseases tend to receive institutional elderly care.

**Conclusions:** In addition to the physical disease itself, elderly people with chronic diseases were often disturbed by psychological problems such as loneliness, anxiety and depression. The recommendations are to encourage the use of electronic health records and follow-up services by a family doctor to meet the needs of the elderly for medical and mental health services nearby, to improve psychological intervention, disease prevention and treatment capabilities, take the realization of “community–family–individual” health management as the main line to promote the construction of diversified elderly care systems such as by combining the advantages of home-based elderly care in spiritual regulation and the advantages of other care modes, to improve the physical and mental health management of chronic diseases through “Internet plus”, to achieve a precise match between supply and demand.

**Acknowledgments**

This study is supported by the National Natural Science Foundation of China “Research on joint evaluation of supply efficiency and integration degree of different home-based elderly care and medical support combination modes” (71804076)

## BEPS23006: APPLICATION OF CLUSTER MANAGEMENT IN PATIENTS WITH PICC WEATHERIZATION

Hengying Che^a^, Xiubin Tao^a^, Zhangbin Ling^a^, Juan Tao^a^, Liang Zhong^a^

^*a*^ *Yijishan Hospital, the First Affiliated Hospital of Wannan Medical College, Wuhu, China.*

**Background:** To explore the effect of cluster management strategy in reducing and preventing catheter related complications in PICC catheterization patients.

**Subjects and Methods:** Patients undergoing PICC intubation in the PICC outpatient department of a third class A hospital from October to December 2019 under the conventional management strategy were selected as the control group, and patients undergoing PICC intubation in the PICC outpatient Department under the cluster management strategy from October to December 2020 were selected as the observation group. The control group obtained relevant data by reviewing patients’ clinical case data, telephone return visits, and inquiring nurses on duty, etc. The observation group obtained relevant research data by specially-assigned person through the table designed in advance after discussion by the research team, and compared the incidence of PICC complications between the two groups.

**Results:** There was no statistical difference in the general information between the two groups (P<0.05). In terms of medical adhesion-related skin mucosal injury, the incidence of observation group (6.2%) was lower than that of control group (9.0%), and there was statistical difference between the two groups (P<0.05). The incidence of device-related stress injury in control group (6.5%) was significantly higher than that in observation group (2.2%), and there was a significant difference between the two groups (P<0.05). The local infection of puncture site in the observation group (4.9%) was significantly lower than that in the control group (9.2%), and there was statistical difference between the two groups (P<0.05). The incidence of bleeding in the observation group (6.1%) was lower than that in the control group (8.6%), and there was a statistical difference between the two groups (P<0.05).

**Conclusions:** Cluster management strategy can improve the success rate of PICC puncture, effectively reduce the occurrence of local skin injury, bleeding, puncture site infection and other related complications, ensure the normal function of PICC catheter, improve patient satisfaction.

**Acknowledgments**

In 2022, Anhui Medical Yijishan Hospital Management and Service Innovation Project CX2022014 Construction and Application Of Intravenous Treatment Quality Sensitivity Index System Based on Risk Management; 2021 Provincial Quality Project of Anhui Universities 2021xnjys032, Virtual Teaching and Research Department of Internal Medicine Nursing.

## BEPS23007: TREATMENT CONCEPT AND TECHNICAL INNOVATION OF GALLSTONE COMBINED WITH EXTRAHEPATIC BILE DUCT STONES

Xiuhan Jiang^a^, Saihua Li^a^, Jianfa Zhang^a^, Hailong Hu^a^, Li Zhang^a^, Chi Cao^a^, Zihan Chen^a^, Peng Li^a^

^*a*^ *Sanmenxia Hospital of the Yellow River, Sanmenxia, Henan 472000, China.*

XJ and SL contributed equally to this study.

**Background:** Choledocholithiasis can be managed by exploration of ductus choledochus for choledocholelithes by choledochofiberoscope via cystic duct, Choledochoscope to explore choledocholithiasis through the common bile duct, T duct drainage or primary suture was used.

**Subjects and Methods:** The aim of this study is to evaluate the safety and effectiveness of common bile duct exploration through the LCBED T duct drainage or primary suture was used. With a specific emphasis on exploration of ductus choledochus for choledocholelithes by choledochofiberoscope via cystic duct, Choledochoscope to explore choledocholithiasis through the common bile duct. Objective to investigate the feasibility, indications and methods of the primary suture of common bile duct after exploration under fibercholedochoscope. Between January 2019 and June 2022, a total of 1226 choledocholithiasis patients accompanied by exploration of ductus choledochus for choledocholelithes by choledochofiberoscope via cystic duct (TC), Choledochoscope to explore choledocholithiasis through the common bile duct, T duct drainage (T-tube) or primary suture was used (LCDBE). Patients were regularly followed up at bimonthly intervals or more frequently in presence of any symptom. Primary outcomes measures included overall operative time, length of hospital stay, and postoperative bile leaks.

**Results:** Successful bile duct clearance was 100 % in the LCBDE group, 7.01 % in the Cystic duct, and 23.16 % in the T duct drainage. 69.82% in the LCBDE Primary suture. There were less bile leaks (>3d) after primary suture was used (LCDBE) (16.59 %) than TC (20.93%) and T-tube (40.49 %), which prolonged hospitalization in the T-tube group more than in the TC and LCBDE groups. For choledocholithiasis patients accompanied by cholecystitis, 2 groups (TC and LCBDE groups) were comparable in operative time. However, for choledocholithiasis patients accompanied T-tube had a significantly longer operative time than the LCBDE group (210.856 p =0.004, P<0.001). By comparing the width of the common bile duct, the width of the gallbladder duct, the time of biliary leakage, the success rate of stone extraction, the presence or absence of biliary residual stone, the method of stone extraction (net basket, irrigation, laser lithotomy), the specifications of suture lines, the suture method, the suture length, the suture margin, the operation time and the hospitalization cost, the individualized best surgical method was obtained.

**Conclusions:** TC appears to be a more effective approach with a lower complication rate and is an easy-to-perform technique that simplifies the procedure by avoiding choledotomy and subsequent T-tube insertion. Primary suture is suitable for most patients with biliary calculi. The occurrence of biliary leakage can be significantly reduced by appropriate suture selection and improvement of suture technique, as well as precise suture length and margin control. T-tube drainage, as a classic biliary exploration operation, is suitable for patients with biliary stricture.

## BEPS23008: APPLICATION AND DISCUSSION OF SIMULATOR TRAINING COMBINED WITH LAPAROSCOPIC SURGERY VIDEO IN CLINICAL TEACHING OF OBSTETRICS AND GYNECOLOGY

Huiqin Liu^a^, Cuilan Yun^a^, Qiqige Xi^b^, Zhigang Yang^c^, Hua Ma^d^, Zhijie Zhang^a^, Liping Zhao^a^, Haifeng Wu^e^, Yaxuan Guo^b^, Xu Yang^b^, Yuemei Zhang^a^, Yu Zhang^b^, Nannan Li^a^, Guangming Bai^b^, Yahua Jia^b^

^*a*^ *Department of Obstetrics and Gynecology, Baotou Central Hospital, Baotou City, 014040, Inner Mongolia Autonomous Region, China,* ^*b*^ *Baotou Clinical Medical College, Inner Mongolia Medical University, Baotou 01040, Inner Mongolia Autonomous Region, China,* ^*c*^ *Urinary Surgery, Baotou Central Hospital, Baotou City, 014040, Inner Mongolia Autonomous Region, China,* ^*d*^ *Supply room, Baotou Central Hospital, Baotou City, 014040, Inner Mongolia Autonomous Region, China,* ^*e*^ *General surgery department, Baotou Central Hospital, Baotou City, 014040, Inner Mongolia Autonomous Region, China.*

**Background:** To investigate the effectiveness of simulator training in the clinical teaching of gynecologic laparoscopic surgery (GLS).e teaching mode of the BoMed era simulator training combined with watching laparoscopic surgery videos can better enable the zero practical training/residents to comprehend the surgical skills, improve the clinical surgical operation, increase the volume and precision of surgery in hospitals, increase the mastery of clinical skills to better accomplish the purpose of teaching and improve the quality of teaching.

**Subjects and Methods:** To investigate the effectiveness of simulator training in the clinical teaching of gynecologic laparoscopic surgery (GLS). Eight third-year registrars/residents who were trained in the laparoscopic simulator as well as watched laparoscopic surgery videos from November 2022 to March 2023 but had zero practical skills were used as study subjects. The experimental group (4) used the new teaching model of laparoscopic surgery video in combination with simulator training in the BoM era, and the control group (4) used the traditional teaching model. At the end of the 5-month training period, the participants of both groups were evaluated and surveyed separately on the practical laparoscopic operations to evaluate the effectiveness of clinical teaching.

**Results:** The total score of minimally invasive surgical operation of students in the experimental group was significantly higher than that of the control group; questionnaires were made in four aspects: hands-on ability, clinical operation ability, learning interest, and self-improvement, and satisfaction rate of students in the experimental group was significantly higher than that of the control group (P<0.05). Conclusion: The teaching mode of the BoMed era simulator training combined with watching laparoscopic surgery videos can better enable the zero practical training/residents to comprehend the surgical skills, improve the clinical surgical operation, increase the volume and precision of surgery in hospitals, increase the mastery of clinical skills to better accomplish the purpose of teaching and improve the quality of teaching.

**Conclusions:** During the teaching period, there was no significant difference between the two groups in terms of the number of procedures they participated in and the length of time they watched laparoscopic videos, and the independent completion of laparoscopic ovarian cystectomy LOC was used as the evaluation criterion.

## BEPS23009: ANALYSIS OF THERAPEUTIC EFFECT OF SEQUENTIAL COMBINATION OF TRADITIONAL CHINESE MEDICINE AND PLATELET RICH PLASMA INJECTION ON REFRACTORY STRESS INJURY

Jiali Huang^a^, Tao Yang^a^, Ming Cheng^a^, Tao Xiang^a^, Shunzhi Zuo^a^, Yunyi Xiao^a^, Long Jia^a^

^*a*^ *Jinniu District People’s Hospital of Chengdu, Chengdu, 610036, China.*

**Background:** To investigate the clinical effect of sequential treatment of traditional Chinese medicine combined with platelet rich plasma (PRP) injection on refractory wounds

**Subjects and Methods:** Ninety patients with refractory stress injury admitted to Jinniu District People’s Hospital from January 2020 to December 2021 were selected and divided into treatment group and control group with 45 cases each according to the SAS software random procedure. The control group was treated with traditional Chinese medicine on the basis of debridement and routine cleaning and dressing change; the treatment group was treated with PRP injection and PRP gel external application on the basis of the control group. The wound healing and pressure injury healing evaluation scale (PUSH) were monitored and recorded on the 3rd, 7th, 10th and 14th days of the two groups of patients, and the growth factors and inflammatory indicators were detected after the treatment.

**Results:** There was no significant difference in PUSH scores between the control group before treatment and the 7th day of treatment (P>0.05), but there was significant difference in PUSH scores between the control group before treatment and the 14th day of treatment (P<0.05); There was no significant difference in PUSH scores between the treatment group before treatment and the 7th day of treatment (P>0.05), but there was significant difference in PUSH scores between the treatment group before treatment and the 14th day of treatment (P<0.01). There was no statistical significance between the treatment group and the control group on the 7th day of each wound score index (P>0.05), and there was a statistical difference between the treatment group and the control group on the 14th day of each wound healing index (P<0.05). VEGF, TGF-B1, EGF, PDGF-BB in the treatment group were significantly different from those in the control group after 7 days of treatment (P<0.005), while VEGF, TGF-B1, EGF, PDGF-BB in the treatment group after 14 days of treatment were significantly different from those in the control group after 14 days of treatment (P<0.001). There were statistically significant differences in IL-6, PCT, CRP between the control group before treatment and the 7th day of treatment (P<0.05), and no statistically significant differences in WBC and NEUT (P>0.05). There were statistically significant differences in all inflammatory indicators between the control group and the 14th day of treatment (P<0.01). There were statistically significant differences in IL-6, PCT, CRP, WBC between the treatment group and the 7th day of treatment (P<0.05), There was no significant difference in NEUT (P>0.05); All inflammatory indexes in the treatment group were significantly different from those before treatment on the 14th day of treatment (P<0.01). Compared with the control group on the 7th day, there were statistically significant differences between the treatment group and the control group in terms of various inflammatory indicators (IL-6, PCT, WBC) (P<0.05), while there were no statistically significant differences between NEUT and CRP (P>0.05). There were statistically significant differences between the treatment group and the control group on the 14th day in terms of various inflammatory indicators (P<0.01).

**Conclusions:** Platelet rich plasma (PRP) combined with traditional Chinese medicine in sequential treatment of refractory pressure injury can reduce inflammatory exudation, accelerate wound convergence, reduce and control inflammatory reaction, promote growth factor release, accelerate wound healing, improve drug synergy, effectively promote the healing of refractory wounds, shorten the healing time, and is a new and effective treatment method for refractory pressure injury.

**Acknowledgments**

This research was supported by Sichuan Medical Association Wound Disease (Taige) Special Research Project (Project No.: 2021TG28)

## BEPS23010: STUDY ON TEACHING STRATEGIES OF CHINESE ORAL COMMUNICATION IN PSYCHOLOGYCAL NURSING FOR NURSING MAJOR IN VOCATIONAL COLLEGES

Ying Song^a^, Yanfang Ran^a^

^*a*^ *Academic Affairs Office, Zhengzhou Railway Vocational and Technical College, Zhengzhou, Henan 450000, China*

**Background:** Vocational colleges are a significant base for talent training in our country, which supplies a large number of talents for the society each year. Nursing is also a popular major in vocational colleges. The future career development of most nursing students is nursing staff. Every year, a large number of students graduate from nursing major of vocational college and join the nursing industry. It is unavoidable for them to communicate with patients and their families in the process of working. Due to the great pain caused by diseases or accidents, patients are very easy to have mental health problems. They often experience anxiety, sensitivity, depression, etc. Thus, nursing staff need to be good at observing words and expressions of patients properly and to have good oral communication ability to ease the emotions of patients and give them necessary support and help.

**Subjects and Methods:** The research object of this paper is the nursing students in vocational colleges. This paper employs research methods such as literature review, observation, and investigation to collect data and analyze the results. On this basis, this paper has an in-depth discussion of nursing staff on how to improve language communication skills, provide high-quality nursing services, better meet the needs and expectations of patients, and further enhance patients’ satisfaction and treatment effect when facing their mental health problems.

**Results:** Chinese oral communication teaching has not got enough attention in nursing education in traditional vocational colleges, which leads to certain problems in oral expression and communication of the students. This requires teachers to change their teaching concepts in time, and guide students to fully recognize the importance of mental health in assisting patients’ treatment. According to the characteristics of students in their own class, teachers need to innovate teaching methods and educational forms to improve the quality of oral communication teaching. Meanwhile, teachers need to adopt effective teaching strategies to improve students’ oral communication ability to lay a good foundation for their future employment.

**Conclusions:** Through Chinese oral communication teaching, nursing students’ observation and communication ability can be improved, which enable them to pay timely attention to the patients’ psychological condition, learn to listen, understand, and respect the feelings of others, and use accurate and standardized language to have in-depth and cordial communication with patients. At the same time, they can take initiative, be comprehensive and act according to the circumstances in the process of completing the nursing work, in order to better care for the physical and mental health of patients.

## BEPS23011: PREPARATION AND PERFORMANCE STUDY OF TUNGSTATE SR_9_LA_2_W_4_O_24_: EU^3+^ PHOSPHOR SUITABLE FOR MEDICAL TESTING

Guo Feng^a^, Weifeng Xie^a^, Feng Jiang^a^, Jianmin Liu^a^, Entao Zheng^a^, Junling Yu^a^, Lidan Wu^b^, Hui Wang^b^, Chuan Shao^c^, Qian Wu^a^, Qing Hu^a^

^*a*^ *Department of Material Science and Engineering, Jingdezhen Ceramic University, Jingdezhen 333000, China,* ^*b*^ *Jingdezhen Oceano Ceramic Company, Jingdezhen 333000, China,* ^*c*^ *Jingdezhen Market Supervision and Management Comprehensive Inspection and Testing Center, Jingdezhen 333000, China.*

**Background:** The rapid development of phosphor application materials and technology provides a very important platform and means for the research and development of medical detection, and the key to effective control and treatment of diseases is that accurate detection can be carried out at an early stage, so the development of new, stable, simple preparation conditions, practical value of red phosphor is a very meaningful work.

**Subjects and Methods:** Red phosphor has excellent optical properties, can emit red fluorescence, has the advantages of high stability and high efficiency, and can be used for biomedical detection. In this paper, a novel Sr_9_La_2_W_4_O_24_: Eu^3+^ red phosphor was prepared by solid phase method at 1050℃ for 2 hours, and the effect of Eu^3+^ ion doping on its performance was systematically studied.

**Results:** The results show that when the doping amount of Eu^3+^ ions *x* ≥ 0.06, the diffraction peak of SrWO_4_ becomes more and more obvious, indicating that Eu^3+^ ions have reached solid solution saturation. The quenching concentration of Sr_9_La_2-*x*_W_4_O_24_: *x*Eu^3+^ phosphor was 0.02, and the critical distance of Eu^3+^ in the Sr_9_La_2_W_4_O_24_ matrix was 2.981 nm. The color coordinates of Sr_9_La_1.96_W_4_O_24_: 0.04Eu^3+^ phosphors are (0.671, 0.329), very close to the red standard color coordinates (0.67, 0.33). The *D*_*50*_ (median particle size) of the phosphor prepared by the solid phase method is 2.43 *μ*m, The use of heat treatment and heterogeneous methods to strengthen the conversion of fluorescent materials in disease and biomedical detection has great significance and considerable application prospects.

**Conclusions:** The phosphor particles prepared by the solid phase method are spherical-shapedwith narrow particle size distribution, some particles are square-like, the particle agglomeration is lighter and soft agglomeration, the edges and surfaces are clear, the grain development is better. Sr_9_La_2_W_4_O_24_: Eu^3+^ red phosphor will become an important tool to assist related medical testing, and is also widely used in portable and real-time related testing, providing important support and guarantee for medical testing.

**Acknowledgments**

This work was supported by the National Natural Science Foundation of China [grant numbers 52072162, 51962014, 52262003, 52362041]; Jiangxi Provincial Natural Science Foundation [grant numbers 20202ACBL214006, 20202ACBL214008, 2020ZDI03004, 20202BABL214013, 20232ACB204012]; the Projects of Jiangxi Provincial Department of Science and Technology (20202BABL214013).

## BEPS23012: SWEET TEMPTATIONS AND HEALTH CONSCIOUSNESS: UNRAVELING THE MOTIVATIONS BEHIND SUGAR REDUCTION BEHAVIOR

Mengqiao Zheng^a^, Weilun Huang^a^, Xuemeng Zhao^a^, Chengsi Zheng^a^

^*a*^ *Department of Finance and International trade, Wenzhou Business College, Wenzhuo City 325000, China*

**Background:** To explore the relationship between individuals’ sugar-reduction behavior and the incentives of expectation, specifically emotional expectancy and physical expectancy, in China. The study also aims to investigate the mediating effects of social relationship stimulus (*SRS*) and sugar-reduced products marketing stimulus (*SMS*), as well as the moderating effects of respondents’ individual characteristics on the motivation-sugar-reduction behavior relationship.

**Subjects and Methods:** An online questionnaire was designed and administered to randomly selected respondents in China. The questionnaire explored various aspects, including respondents’ motivation for sugar reduction, their experiences with sugar reduction, their recognition of the health risks associated with excessive sugar consumption, and demographic variables such as gender, age, education, annual income, and residential city. A total of 597 valid responses were collected and analyzed using structural equation modeling.

**Results:** The findings indicate that respondents’ primary motivation for adopting sugar-reduction behavior is driven by physical reasons. The mediating role of sugar-reduced products marketing stimulus (*SMS*), particularly through food package labels and social media advertisements, has a more significant influence on the relationship between motivation and sugar-reduction behavior compared to social relationship stimulus (*SRS*). However, recommendations from friends as *SRS* also have a considerable moderating impact. Moreover, respondents’ individual characteristics, such as their experience with sugar reduction, recognition of the health effects of sugar consumption, age, gender, education, annual income, and residential city, exhibit varying degrees of moderating influence on these relationships.

**Conclusions:** This study highlights the critical role of physical incentives in driving individuals’ motivation to reduce their sugar intake and adopt healthy dietary behaviors in China. Sugar-reduced products marketing stimulus, particularly through labeling and social media advertisements, plays a crucial role in influencing individuals’ sugar-reduction behavior. Recommendations from friends, as form of social relationship stimulus, also have a significant impact on individuals’ sugar-reduction behavior. The study further underscores the importance of considering individual characteristics when developing personalized interventions to promote healthy dietary behaviors and reduce the negative health impacts of excessive sugar consumption. These insights can inform policymakers, marketers, and healthcare professionals in developing targeted interventions to promote healthy dietary behaviors and address the challenges posed by excessive sugar consumption in China.

## BEPS23013: RESEARCH ON THE PSYCHOLOGICAL STRATEGIES OF COMMUNITY COPING WITH PUBLIC PANIC IN EMERGENCY PUBLIC CRISIS: COMBINING EPIDEMIC PREVENTION AND CONTROL IN SHENYANG

Tao Song^a^, Zhilin Zhuang^b^

^*a*^ *Department of Information Engineering, Liaoning Economic Vocational Technological Institute, Shenyang 110122, China,* ^*b*^ *China University of Political Science and Law, Beijing 102249, China.*

**Background:** Sudden public crisis events not only affect the daily production and life of community people, but also have a huge impact on their psychology. Based on the background of epidemic prevention and control, it is found that community residents will experience anxiety in sudden public crisis events., panic and other psychological pressures require community workers to serve the psychological and emotional stability of residents, explore the panic situation of community people in the handling of sudden public crisis events, and provide reference for the mental health prevention and control of the community in the future.

**Subjects and Methods:** Through the empirical research method of community observation and in-depth interviews, this paper deeply studies the current situation of community participation in public crisis management and epidemic prevention and control management, and analyzes the problems and causes of panic among community residents.

**Results:** In sudden public crises, grassroots community workers have a heavy psychological burden due to heavy work, the degree of intelligence in community work is not high, the information release is not timely and accurate, and the community people’s confidence in the effectiveness of grassroots epidemic prevention measures is insufficient. Problems such as lack of confidence will lead to panic among the people. In response to these problems, strategies to serve the mental health of the people are put forward, the service system is improved, and the basic living security system of the people is established and improved. Improve the complete community wisdom platform, convey real information to residents in a timely and effective manner, and enhance the public’s sense of trust. The comprehensive and collaborative development of community governance allows more volunteers to join the ranks of community workers and enrich grassroots forces, thereby eliminating public panic and maintaining social security and stability.

**Conclusions:** Facing the problem of how the community responds to the panic psychology of the people in a crisis event, the community can adopt the strategy of improving the crisis service guarantee system and establishing a smart information exchange platform, and the community staff organize the people to share social responsibilities and service obligations. These methods achieve flattening of community organizations, efficient crisis management, and comprehensive governance. These methods can effectively help residents overcome psychological panic during sudden crisis events, alleviate the impact of negative emotions, build confidence in overcoming difficulties, and also provide reference experience for social psychological services in other similar public crisis events.

## BEPS23014: RESEARCH ON MEDICAL IMAGE RECOGNITION ALGORITHM OF COVID-19 BASED ON DEEP LEARNING

Jinlan Guan^a^, Jiequan Ou^b^, Guirong He^c^, Huimin Guo^d^, Guanghua Liu^a^

^*a*^ *Guangdong AIB Polytechnic, Guangzhou 510507, China,* ^*b*^ *Guangzhou Light Industry School, Guangzhou 510650, China,* ^*c*^ *Zhongshan Torch Polytechnic, Zhongshan 528437, China,* ^*d*^ *Guangdong Peizheng College, Guangzhou 510830, China.*

**Background:** The traditional detection method of coronavirus is nucleic acid detection, but it is inefficient and costs a lot of manpower and material resources. In order to effectively control the spread of the epidemic, the medical community needs to rapidly and accurately diagnose and treat covid-19 pneumonia. At present, lung CT scan has become one of the important methods for diagnosing covid-19 pneumonia, for it can provide high-resolution images of the lung to help doctors diagnose and treat patients. The classification and segmentation of the lung images of patients with covid-19 pneumonia based on deep learning algorithm can improve the diagnostic efficiency. In order to compare the classification and segmentation effects of the existing deep learning algorithms on the images of patients with covid-19, this paper selects three algorithms for comparative study. They are ResNet-50, Inception-v3 and U-Net, which are three famous deep learning algorithms.

**Subjects and Methods:** Based on the deep learning algorithm, ResNet-50, Inception-v3 and U-Net were selected to classify and segment the lung images of the novel coronavirus pneumonia, and the accuracy and loss rate of the model were obtained. By doing this, we can compare the three algorithms which is more suitable for the study of the patients with covid-19 pneumonia image recognition, improve the efficiency of clinical diagnosis.

**Results:** The comparison and analysis of the three algorithms shows that the classification accuracy of the three algorithms is: ResNet-50:92.5%, Inception-v3:94.0% and U-Net:91.0%, and the segmentation accuracy of the three algorithms is ResNet-50:75.0%, Inception-v3:78.0% and U-Net:85.0%.

**Conclusions:** Therefore, the Inception-v3 algor-

ithm is superior in classification, while U-Net algorithm has obvious advantage in segmentation model.

**Acknowledgments**

This work was supported by a project grant from Scientific Research Platform and Project of Department of Education of Guangdong Province (New Generation Information Technology Project) “Research on Medical Image Diagnosis Algorithm of COVID-19 Based on Deep Learning” (Project No.: 2021ZDZX1125) and Key Scientific and Research Platforms and Projects of Colleges and Universities in Guangdong Province (Youth Innovative Talents Project) “Research on the Model of Considering Secondary Infection with COVID-19” (Project No.: 2021KQNCX133)

## BEPS23015: CLINICAL AND MRI ANALYSIS OF HYPERTROPHIC INFERIOR OLIVARY DEGENERATION

Mengqiu Liu^a^, Feiyan Zeng^a^, Fangfang Chen^a^, Lei Yuan^a^, Ying Liu^a^

^*a*^ *Department Radiology, The First Affiliated Hospital of University of Science and Technology of China, Hefei 230001, China.*

**Background:** To summarize the clinical and MRI manifestations of patients with hypertrophic olivary degeneration (HOD) and the causes of HOD.

**Subjects and Methods:** The clinical and MRI features of 15 patients with HOD were analyzed retrospectively. All patients underwent 3.0T MRI examination, with scanning sequences including T1 weighting imaging, T2 weighting imaging and T2-FLAIR sequences.

**Results:** The clinical manifestations of the 15 patients were as follows: 2 patients had soft palate tremor, 3 patients had unilateral limb tremor, 4 patients had nystagmus, 5 patients had weakened pharyngeal reflex, 3 patients had cough due to drinking water, 2 patients had walking instability, 1 patient had unclear speech, 1 patient had sudden hoarseness, 2 patients had blurred vision, and 2 patients had no special clinical symptoms. Among the 15 patients, 9 had HOD due to previous brainstem hemorrhage, 3 had brainstem infarction, 1 had cerebellar hemorrhage, 1 had brainstem cavernous hemangioma, and 1 had contusion and laceration caused by left thalamus and brainstem trauma. The average course of HOD was 13.6 ± 9.3 months. 11 cases showed bilateral HOD on MRI, 3 cases showed right HOD, and 1 case showed left HOD. All cases followed the qualified Guillain–Mollaret pathway conduction pattern. MRI showed slightly high and high signal intensity on T2WI with clear or blurred boundaries. Among the 3 follow-up cases, case 1 had a course of disease from 8 to 10 months and the degree of inferior olive nucleus hypertrophy gradually weakened; Case 2 has a course of disease from 22 months to 31 months, and the inferior olive nucleus gradually atrophies; Case 3 has a course of 32 to 35 months, and the degree of hypertrophy of the lower olive nucleus worsens.

**Conclusions:** HOD is often secondary to lesions in the dentate nucleus red nucleus inferior olivary nucleus circuit. The etiology of this group of cases is mostly posterior circulation vascular lesions, and no clear correlation between HOD and disease course was found in this follow-up case. When discovering typical MRI characteristic signals in the olive nucleus region, it is necessary to actively search for bleeding or infarction in the posterior circulation supply area to support the diagnosis of HOD and avoid misdiagnosis.

**Acknowledgments**

This research is supported by the Bethune Charitable Foundation (Fund number: bqe-tzb-2019-007).

## BEPS23016: ANILINE AND NITROBENZENE COMPOUNDS WERE DETERMINED SIMULTANEOUSLY BY ASE-GC-MS IN THE PREVENTION OF OCCUPATIONAL HAZARDS

Qiang Zhang^a^, Xiuwen Zhang^b^, Dawei Xiao^a^, Chuang Zhao^b^, Jianling Bi^b^, Kai Liu^a^, Rong Wang^a^, Weiwei Shi^a^

^*a*^ *Center for Soil Pollution Control of Shandong, Jinan, 250012, China,* ^*b*^ *Shandong Institute of Geophysical and Geochemical Exploration, Jinan, 250013, China.*

**Background:** Aniline is a colorless oily liquid with a strong odor, which turns brown when exposed to air or sunlight and is slightly soluble in water. Pure nitrobenzene is a colorless to light yellow oily liquid with a special almond oil-like smell and is almost insoluble in water. Both are miscible with ethanol, ethyl ether, or benzene. In industrial production such as dyes, pharmaceuticals, rubber, paints, and plastics, inhaling high concentrations of these compounds and direct or indirect skin contamination can lead to poisoning, a common occupational disease. Acute poisoning often occurs during or a few hours after work. Regular determination of the concentrations of aniline and nitrobenzene compounds in the work environment can provide precise evaluations for occupational exposure to these toxic substances, which is beneficial for keeping the exposure risk to acceptable levels and protecting human health.

**Subjects and Methods:** To achieve synchronous detection of aniline and nitrobenzene compounds in occupational disease prevention, through the regulation of pH and optimization of extraction conditions, the determination of aniline, 4-nitroaniline, 2,4,6-trichloroaniline, 3,4-dichloroaniline, 4-chloro-2-nitroaniline, 2,6-dichloro-4-nitroaniline, nitrobenzene, o-nitrotoluene, 2,6-dinitrotoluene, 2,4-dinitrotoluene, 2,4-dinitrochlorobenzene, 2,4,6-trinitrotoluene, 12 types of aniline and nitrobenzene compounds was realized using gas chromatography-mass spectrometry (GC-MS). During extraction in the sample, 150 μL of ammonia was added to adjust the soil pH, acetone-n-hexane (1 + 1) was used as an extraction solvent, and after vacuum concentration and purification, GC-MS was used for determination, supplementing the deficiencies of existing standards.

**Results:** The experimental results showed that under optimized conditions, six types of aniline compounds and six types of nitrobenzene compounds had a good linear relationship in the 1～10 mg/L range, with correlation coefficients R all >0.99. When the sampling amount was 20.00 g, the detection limits of the 12 compounds were between 0.02～0.05 mg/kg. The recovery rate of the actual soil sample was between 78.4 %～92.1 %, and the precision (RSD) was all <10%. Good linearity and high precision can achieve rapid synchronous detection in the prevention of occupational hazards.

**Conclusions:** This method is applicable for the simultaneous detection of various aniline and nitrobenzene compounds in the prevention of occupational hazards. It has a good determination concentration linearity and high precision, can provide a more accurate and reliable detection method in occupational disease prevention, and provide a certain risk assessment basis for exposure to aniline and nitrobenzene compounds under occupational exposure conditions.

**Acknowledgments**

This project is supported by the fund project (Scientific research project of the Shandong Provincial Geological and Mineral Exploration and Development Bureau + Experimental research on the determination of aniline and nitrobenzene compounds in soil by pressurized fluid extraction-gas chromatography-mass spectrometry method + KY202109).

## BEPS23017: A STUDY ON RESOURCE ALLOCATION OF HOSPITAL DAY SURGERY BEDS BASED ON M/M/C QUEUEING MODEL

Lan Huang^a^, Chuan Zhang^a^, Fang Tan^a^, Yang Chen^a^, Jianjun Jiang^a^, Tao Li^b^, Shan Chen^a^

^*a*^ *Department of Palliative Medicine, West China School of Public Health and West China Fourth Hospital, Sichuan University, Chengdu, 610044, China,* ^*b*^ *Department of Stomatology, Qujing No.1 Hospital, Qujing, 202150, China.*

**Background:** This study aims to explore the scientific and reasonable allocation plan of day surgery bed resources in view of the problems of inefficient input and output of bed resources due to managers’ extensive allocation of day surgery bed resources. Scientific and reasonable allocation of hospital bed resources for day surgery can effectively shorten the waiting time of patients and provide theoretical and practical basis for the healthy and orderly development of day surgery in China.

**Subjects and Methods:** This paper is to scientifically and rationally allocate bed resources for day surgery, with the optimization goal of effectively reducing the average waiting time of patients, the team of day surgery bed resources for allocation. This paper took the annual query data of urological surgery in Hospital H in 2019 as the empirical case object. The data fields include patient gender, age, department, bed, surgery start date, surgery start time, surgery end date, surgery end time, patient departure date, patient departure time, surgery type, and discharge date, etc. By formulating the M/M/C queueing model, the day surgery bed resources were allocated with a view to minimizing the average number of waiting patients and maximizing the utilization rate of bed resources.

**Results:** The optimal number of day surgery beds to be allocated in the urology department of Hospital H should be 5 beds. The impact on the bed resource allocation results was analyzed by considering fluctuations in patient arrival and length of stay. The current bed resource allocation strategy in the urology department of Hospital H was comparatively analyzed. The research results showed that the average utilization rate of bed resources increased by 11.9% and the number of served patients increased by 249, which was significantly superior compared to the current day surgery bed resource allocation strategy in the urology department of Hospital H.

**Conclusions:** Based on the M/M/C queueing model analysis, this paper can provide a theoretical basis and practical reference for the scientific and reasonable allocation of day surgery bed resources in current healthcare institutions, and provide theoretical and practical basis for the healthy and orderly development of day surgery in China.

**Acknowledgments**

This work was supported by National Natural Science Foundation of China Research Project (Grant No. 71804117). Ethics Approval: Management of “hospital-community” transitional care network for patients with day surgery and development path (West China Hospital Biomedical Ethics Committee 2020 No.76).

## BEPS23018: THE IMPACT OF PSYCHOLOGICAL NURSING INTERVENTION IN PERIOPERATIVE PERIOD ON THE LEVEL OF DEPRESSION AND ANXIETY AND THE PSYCHOLOGICAL STRESS REACTION OF THE PATIENTS WITH AUTOGENOUS CRANIOPLASTY

Yaohui Peng^a^, Yongjuan Feng^b^, Jing Hou^a^, Huiling Wang^a^, Lijuan Li^a^

^*a*^ *Department of Neurosurgery, Guang’an People’s Hospital, Guang’an, Sichuan, China,* ^*b*^ *Department of Infectious Disease, Guang’an People’s Hospital, Guang’an, Sichuan, China.*

**Background:** In order to provide certain theoretical reference for the clinical treatment, the paper explores the impact of psychological nursing intervention in perioperative period on depression and anxiety and psychological stress reaction of the patients with autogenous cranioplasty.

**Subjects and Methods:** 42 patients with the treatment autogenous cranioplasty in our hospital from May 2018 to January 2020 are selected in the paper, and they are divided into 21 cases of control group and observation group respectively. The control group is adopted with the ordinary nursing intervention in perioperative period, while the observation group would be enhanced with the psychological nursing intervention of patients based on that of the control group, so that there would be comparison of the anxiety and depression of the two groups and their own psychological stress response index and nursing compliance.

**Results:** The self-rating anxiety scale (SAS) and the self-rating depression scale (SDS) of the observation group after the intervention are lower than those before the intervention and those of the control group (P < 0.05). After the intervention, the systolic blood pressure (SBP), diastolic blood pressure (DBP) and heart rate (HR) of observation group is lower than those before the intervention and those of the control group (P < 0.05). The excellent-and-good rate of nursing compliance of patients in observation group is 90.48%, while that of the patients in control group is 61.91%. The observation group is significantly higher than the control group (P<0.05).

**Conclusions:** In the perioperative period of autogenous cranioplasty, focusing and enhancing the psychological nursing intervention of patients would improve the anxiety and depression of the patients and reduces their psychological stress reaction; and it is helpful to improve their nursing compliance to positively work with the operation. Hence, it should be extensively presented and applied in the clinical treatment.

## BEPS23019: EMPIRICAL STUDY ON THE HIGH-QUALITY DEVELOPMENT PLAN OF TERTIARY PUBLIC HOSPITALS

Weiwei Xiong^a^, Jing Guo^a^, Dongmei Li^a^, Liming Jiang^a^, Tong Guan^a^, Shi Yan^a^, Fengqiang Li^a^

^*a*^ *Qianwei Hospital of Jilin Province, Changchun, Jilin 130012, China.*

**Background:** Since 2018, National Health Commission of the People’s Republic of China has established 55 standardized, standardized, and homogeneous evaluation indicators for the functional positioning of tertiary public hospitals, the quality and efficiency of medical services, and the guiding direction of medical reform in public hospitals. By evaluating the development of hospitals each year, it promotes the return of hospitals to public welfare attributes, meets patients’ psychological needs for the public medical system, avoids anxiety and depression caused by excessive medical treatment or poor medical quality, improves patients’ sense of access to medical and health resources, and provides higher quality, fairer, and safer medical and health services for the majority of patients, leading the high-quality development of hospitals.

**Subjects and Methods:** This study aims to comprehensively analyze the connotations and interrelationships of various indicators from the perspectives of medical quality, operational efficiency, sustainable development, and satisfaction, in conjunction with the actual situation of the hospital. It intends to develop a complete improvement plan and integrate it into daily operational management for validation. This will promote the hospital to update management concepts, improve medical behavior, enhance service levels, and drive the ‘three transformations’ and ‘three improvements’, ultimately guiding the high-quality development of the hospital.

**Results:** After implementing improvement measures, compared to 2019, in 2021, the proportion of surgical discharges in the hospital increased by 6.12%, the proportion of level four surgeries among discharged patients increased by 4.73%, the surgical site infection rate decreased by 0.15%, and the mortality rate of low-risk cases decreased by 0.04%. At the same time, the proportion of medical service revenue increased by 1.45%, the average cost per hospitalization decreased by 2.01%, patient satisfaction with medical care exceeded the provincial average and showed a slight improvement, and employee satisfaction with the hospital also showed a slight improvement. Throughout 2021, there were no incidents of patients experiencing accidents due to mental illness.

**Conclusions:** After implementing an improvement plan for one cycle, the hospital has made significant progress in terms of management science, standardization, and lean management, effectively driving technological transformation in the hospital and reflecting the functional positioning of tertiary public hospitals. This has improved medical efficiency, enhanced the motivation of medical staff, strengthened talent development, improved the quality of medical services, promoted patient psychological well-being, increased patient satisfaction, and ensured the high-quality development of the hospital.

**Acknowledgments**

This work was supported by Health Commission of Jilin Province Technology Capacity Enhancement Project (2022GL002).

## BEPS23020: ANALYSIS OF GOLD STANDARDS OF BIOMECHANICS PARAMETERS FOR LW4X ELITE ROWERS WITH 33.6 STR/MIN

Weiliang Lin^a^, Dan Sun^a^, Weidong Liang^a^, Jiasheng Hong^a^, Yongjian Lan^a^

^*a*^ *School of Physical Education and Sports Science, Guangzhou University, Guangzhou, Guangdong 510000, China.*

**Background:** To investigate the application effect of human and boat on 2000m water simulation game of 33.6 str/min for women’s lightweight 4 people double sculls rowers (LW4X) by biomechanics supervisory control system.

**Subjects and Methods:** On March 11, 2013, take 4 elite female lightweight quadruple sculls rowers from Guangdong Rowing Training Base as study object in China, analyze the biomechanical parameters of human and boat in the 2000m water simulation game with 33.6 str/min. The research methods include literature study, expert interview, test and mathematical statistics.

**Results:** First, the rowers’ actual data from catch water angle (maximum 115.3%, minimum 48.7%, average 74.9%), catch slip angle (maximum 74.8, minimum 16.6%, average 44.4%), release slip angle (maximum -16.7%, minimum -4.6%, average -9.0), average force/max. force ratio (maximum 7.7%, minimum -6.4%, average 2.5%), peak force at (maximum 51.4%, minimum -34.1%, average 3.2%), which are higher than that of the target gold standards. Second, their actual indexes such as time over 2000m water rowing (-4.2%), prognostic speed at target rate (effective work per stroke: -1.9%), racing stroke rate (-6.6%), the total angle of per stroke (maximum -4.5%, minimum -0.5%, average -2.95), rowing power (maximum -31.1%, minimum -13.7%, average -23.27), finish release water angle of per stroke (maximum -16.7%, minimum -4.6%, average -9.0%), effective angle (maximum -16.5%, minimum -11.4%, average -14.0%), force max. (maximum -31.8%, minimum -14.8%, average -22.42), force up to 70% max. force angle (maximum -9.2%, minimum -29.2%, average 10%), force down from 70% max force (maximum 29.8.7%, minimum -9.6%, average 2.8%), legs drive distance (maximum 5.4%, minimum -3.0%, average -0.07%), and legs max. speed (maximum -16.7%, minimum -2.9%, average -10.32%), which are smaller than that of gold standard data. Third, the LW4X rowers have significant differences in synchronous power parameters, the actual speed and power effect of the boat is affected, it cannot reach the target golden value from the international top rowing level for LW4X rowers.

**Conclusions:** This research is applicable to rowers’ biomechanical Parameter Monitoring for LM4X in 2000m water simulated race, the rowers’ force patterns of body joints and the optimization Mode of boat’s effective speed and power need to be in-depth research, so that the efficiency parameter system from the cooperation of between rowers and human & boat could be promoted.

**Acknowledgments**

This paper was one of the phased results of monitoring the core competitive ability training of lightweight rowers program funded by Guangdong Provincial Philosophy and Social Sciences Planning Project in 2020(code: GD20HTY02), please allow me to express my gratitude to Guangdong Boat Training Base and Guangzhou University, it was their help specially Coaches Ma Qun, Ji Renxu, Dong Wenfen and other the related students in research team, that made sure this research can be carried out successfully.

## BEPS23021: THE EFFECT OF 8 WEEKS OF BOXING EXERCISE ON THE EMOTIONAL CONTROL OF COLLEGE STUDENTS

Xin Chen^a^, Jun Zhao^a^, Xiangui Bu^a^

^*a*^ *Shandong Sport University, Shandong 250102, China.*

**Background:** With 40 college students as the research subjects, we investigated the effect of boxing exercise intervention on the emotional control of college student groups through 8 weeks of boxing practice.

**Subjects and Methods:** 40 college students with low emotional control ability were screened as experimental subjects through the Courtauld Emotional Control Scale, and expert interview, questionnaire survey, and experimental method were used to carry out 8-week experimental interventions on the 40 college students.

**Results:** 1. Intergroup comparison of emotional control of college students in the experimental and control groups before and after the experiment found that there was a significant difference between the emotional control of male students in the experimental group and the control group after 4 weeks of the experiment (P < 0.05), of which there was a significant difference in anger control (P < 0.05), and there was a significant difference between the emotional control of male students in the experimental group and the control group and the dimensions of each dimension after 8 weeks (P < 0.05); there were significant differences in emotional control of female students in the experimental group after 4 weeks of the experiment and Control group girls anger control and depression control there is a significant difference (P < 0.05)), after 8 weeks of the experimental group and the control group girls emotional control there is a highly significant difference (P < 0.001), including depression control there is a significant difference (P < 0.05), anger, anxiety control there is a highly significant difference (P < 0.001).2. Comparison of the emotional control of the experimental group of boys and girls before and after the experiment found that there was no significant difference in the emotional control of the experimental group of boys and girls and the dimensions of the experimental group after 4 weeks of the experiment (P>0.05), and there was a significant difference in the control of anger and anxiety in the experimental group of boys and girls after 8 weeks (P<0.05).

**Conclusions:** 8 weeks of boxing practice has a positive effect on improving the emotional control of college students and contributes to the control of anger, depression and anxiety. The magnitude of improvement of depression control and anxiety control by boxing practice is related to the length of practice, and a short period of boxing practice has less effect on depression control and anxiety control, and there are gender differences in anger control and anxiety control of college students.

## BEPS23022: ANALYSIS OF THE GUIDANCE OF HIGHER VOCATIONAL PHYSICAL EDUCATION TEACHING ON STUDENTS’ MENTAL HEALTH

Wenbin Wu^a^, Ying Zhou^a^

^*a*^ *Chengde College of Applied Technology, Chengde, 067000, China.*

**Background:** With the progress of science and technology and social development, the demand for talents in modern society is getting higher and higher, and high-quality talents have become an indispensable and important resource in modern society, and higher requirements have been put forward for talents. Higher vocational colleges and universities are responsible for the important mission of cultivating builders and successors of socialism with Chinese characteristics. Healthy mental quality and strong physical fitness are the most basic requirements for qualified talents, and the two are complementary to each other.

**Subjects and Methods:** At present, higher vocational students are facing great academic pressure and employment pressure, and many of them show various mental health problems. Higher vocational colleges and universities have the important responsibility of guiding students’ mental health, and they can achieve the goal of higher vocational teaching in a reasonable way. Firstly, to create a good campus sports culture atmosphere, higher vocational colleges and universities can create a unique campus sports culture through sports competitions and special lectures, etc. Secondly, to establish a trusting and harmonious teacher-student relationship, equal and harmonious teacher-student relationship deeply affects the students’ inner world, and can promote the students’ psychology towards a positive and healthy direction; thirdly, to carefully design the teaching content, highlighting the mental health, higher vocational teachers should make a reasonable design according to the teaching objectives when preparing the lessons. Third, carefully design the teaching content, highlighting mental health, senior teachers in the preparation of lessons, according to the teaching objectives to make reasonable trade-offs, organic integration of mental health content; Fourth, the creation of a combination of engineering teaching mode, through the design of sports training programmes in line with the needs of job practice, to alleviate the pressure on the students; Fifth, to encourage students to explore independently and boldly try to physical education teachers should be actively encouraged to have fear of students should be actively encouraged, to guide and encourage them to boldly try to explore the physical education knowledge. knowledge.

**Results:** Comprehensive physical education teaching is of great significance in cultivating the mental health of higher vocational students. In recent years, many higher vocational colleges and universities have been trying hard to use psychological guidance to carry out physical education teaching and have achieved certain results.

**Conclusions:** In the current higher vocational physical education classes, physical education teachers should use effective guidance to strengthen students’ mental health education and cultivate the healthy mental state of higher vocational students.

## BEPS23023: THE THOUGHT OF “PREVENTIVE TREATMENT FOR DISEASE” AND ITS CLINICAL APPLICATION IN PEDIATRICS OF TRADITIONAL CHINESE MEDICINE

Dongmei Li^a^, Hongyan Shen^b^

^*a*^ *Affiliated Hospital of Liaoning University of Traditional Chinese Medicine, Shenyang, 110032, China,* ^*b*^ *Liaoning University of Traditional Chinese Medicine, Shenyang 110032, China.*DL and HS contributed equally to this study.

**Background:** To explore the theoretical meaning of traditional Chinese medicine (TCM) “preventive treatment for disease” in the classic TCM book Huang Di Nei Jing (Huangdi’s Canon of Medicine), its clinical application scope and social economic value in contemporary TCM pediatrics.

**Subjects and Methods:** In this paper, the author deeply analyzed the theoretical meaning of “preventive treatment of disease” in traditional Chinese medicine by collecting, sorting and studying the literature of ancient Chinese medicine and contemporary relevant works, journals and other literature. At the same time, combined with his own clinical practice, taking “influenza” “asthma” and other common pediatric diseases as examples, the author applied the “preventive treatment of disease” theory to the prevention and treatment of children’s clinical diseases in different periods. To explore the application scope and value of preventive treatment in pediatrics of traditional Chinese medicine (TCM).

**Results:** The theory of preventive treatment for disease embodies the essence of the thought of prevention and health care in the traditional Chinese medicine classic Nei Jing, and has important guiding significance for the clinical practice of pediatrics of traditional Chinese medicine. Its four meanings and theoretical thoughts of “prevention before disease”, “early adjustment for disease”, “prevention against change after disease”, and “prevention and recovery after disease” run through the prevention and treatment of various diseases in pediatrics. Based on the idea of “preventive treatment for disease”, a variety of prevention and treatment interventions are commonly used in pediatric clinical practice, such as bathing method, olfactory and nasal method, aromatherapy or fragrance dressing method, acupoint application method, and chiropractic therapy.

**Conclusions:** The thought of “preventive treatment of disease” reflects the thought of “human oriented” in traditional Chinese medicine -- “unity of nature and man”. The thought of “preventive treatment of disease” has been running through the medical practice of traditional Chinese medicine for thousands of years. It is the core content of the health culture of traditional Chinese medicine, and has important guiding significance for the prevention and treatment of clinical diseases in pediatrics of traditional Chinese medicine, with high social and economic value. The idea of “preventive treatment for disease” should run through the clinical prevention and treatment of diseases in pediatrics.

**Acknowledgments**

This paper was supported by the basic research project of the education department of Liaoning province (2100221274) and the fifth batch of National TCM Clinical Excellent Talents Research Project (TCM Teaching Letter [2022] No.1)

## BEPS23024: EFFECT OF PSYCHOLOGICAL CRISIS INTERVENTION ON INVESTOR SENTIMENT, SUBJECTIVE BELIEF ADJUSTMENT, AND MARKET VOLATILITY

Saiwen Li ^a^

^*a*^ *Lingnan University, Hongkong, China.*

**Background:** This study aims to investigate the effect of psychological crisis intervention on investor sentiment, subjective belief adjustment, and market volatility.

**Subjects and Methods:** 148 market analysts from major financial institutions were randomly selected. Seven days before and 7 days after psychological crisis intervention treatment, a general self-made survey questionnaire, a Self Rating Anxiety Scale (SAS), and a Self Rating Depression Scale (SDS) were administered for evaluation. After the initial assessment, corresponding emergency psychological crisis interventions (including techniques such as venting, counseling, explanation, and cognitive reconstruction) are given.

**Results:** After implementing emergency psychological crisis intervention, the SAS, SDS total rough scores, and standard scores of financial institution market analysts significantly decreased (p<0.01). In the study of financial market anomalies, behavioral finance focuses on investment entities as the research object, integrating traditional financial asset pricing theories based on mathematical models, and introducing the influence of investors’ beliefs, emotions, behavior, and other factors on asset pricing. Investor beliefs, as a subjective probability or expectation, are influenced by investors’ own characteristics and external information shocks. Through the processing of information through Bayesian learning, investors continuously adjust their beliefs, resulting in a distinction between prior and posterior beliefs. The continuous changes in beliefs reflect changes in investor sentiment in the market and ultimately affect the choice of investment decisions. Through this process, investors’ beliefs act on the market, and market yield fluctuations will react on beliefs, thereby achieving a transmission mechanism where information shocks lead to changes in investors’ beliefs, emotional fluctuations, and reactions to the market.

**Conclusions:** This paper demonstrates the inherent mechanism between investor sentiment, subjective belief adjustment, and market volatility by constructing a mathematical model. During the research process, subjective belief variables were introduced. Analyze investor sentiment and market volatility mechanisms through theoretical modeling and empirical research. This paper describes the abnormal volatility path of the market caused by subjective belief adjustment. The logical analysis framework of “belief adjustment → investor sentiment → market volatility” has been established. In empirical research. This paper uses principal component analysis to establish an investor sentiment index, and uses multiple regression and impulse response functions to test the relationship between investor sentiment, subjective beliefs, and stock market volatility. This paper conducts differentiated research based on different styles and industries of assets. The empirical results indicate that investor sentiment has a positive impact on beliefs, and different information preferences will lead to different frequency of emotional fluctuations. The preference for fundamental information is often more conducive to emotional stability. Investor sentiment has a significant positive impact on market returns and volatility. Meanwhile, from an asset style perspective, large cap stocks, low P/E ratios, and blue chip stocks are more susceptible to investor sentiment.

## BEPS23025: CONCERN ON HEALTHCARE INFORMATION SECURITY: IMPROVING AWARENESS OF AND RESPONSE TO SOCIAL ENGINEERING

Huayan Li ^a^

^*a*^ *The Second Affiliated Hospital of Guangzhou University of Chinese Medicine, Guangzhou, Guangdong, China.*

**Background:** In the context of the information age, the healthcare industry is constantly achieving greater alignment with IT development, and a growing number of hospitals are deploying various business systems to improve operational efficiency and service quality. With the increasing use in hospitals, these systems are facing the thread from hackers with unlawful purposes on daily basis. Different from conventional cyber-attacks, social engineering is a hacking technique that targets systems users, and tend to be not only easily overlooked, but also hard to be prevented by hospitals.

**Subjects and Methods:** By emphasizing healthcare information security awareness and sorting out the process and characteristics of social engineering, the author gives suggestions for preventing social engineeringat both the individual and hospital levels

**Results:** The suggestions can significantly improve the healthcare industry’s awareness and response capabilities in this area.

**Conclusions:** In the context of the information age, systems in hospitals often face cyber-attacks. This article analyzes the process and characteristics of social engineering and proposes suggestions on how to prevent social engineering at the individual and hospital levels, in order to enhance the healthcare industry’s awareness of social engineering.

## BEPS23026: STUDY ON THE IMPACT MODEL OF COMMUNITY CHILDREN’S MEDICAL CLINIC BUILDING UNDER THE GUIDANCE OF HEALTHY COMMUNITY

Wei Zhang^a,b,c^, Mengyao Wang^a^, Tianyu Zhang^a^, Bingqian Niu^a^, Runzi Fu^a^, Mengmiao Zhang^a^, Hao Luo^a^, Chengjun Tang^a^, Guangyu Li^a^

^*a*^ *School of Architecture and Planning, Hunan University, Changsha, 410082, China,* ^*b*^ *Hunan Provincial Key Laboratory of Urban and Rural Human Settlements in Hilly Area, Changsha, 410082, China,* ^*c*^ *Hunan International Innovation Cooperation Base on Science and Technology of Local Architecture, Changsha, 410082, China.*

**Background:** The existing community children’s medical buildings are low in infant suitability, and there are widespread problems such as low accessibility, poor comfort, noise and sight interference, etc., resulting in insufficient response, communication and care of patients during the process of seeking medical treatment, anxiety and excessive pressure. In order to meet the requirements of children’s medical building standards for more specialized children, detailed function and community, it is the goal of this study to construct a guiding impact model for the whole life cycle of buildings.

**Subjects and Methods:** In order to build a comprehensive and appropriate impact model for community children’s clinics, this study first established a comprehensive and reasonable index by using NVivo12 qualitative text analysis method to process the existing international and domestic guidelines, and established a preliminary building impact model, obtained a “target layer-criterion layer-indicator layer”. Then the Delphi expert research method was used to remove the most important indexes, and then the analytic hierarchy process (AHP) was used to assign weight to the indexes. The evaluation system takes healthy community as the guideline to build 5 target levels, and subdivided into 13 factors, such as safety and children’s environmental psychology. According to the 13 factors, the relevant guidelines of healthy community and the children’s medical buildings are coded and summarized, and 68 specific indexes are obtained. Finally, the three levels are respectively weighted to construct the children’s community clinic building impact model with weighted values.

**Results:** The model of this study shows that: 1. Among the community factors, the weight of “spatial arrangement” of criterion level is 45.08%, among which “clear circulation lines in the building”, “reducing the traffic distance of indoor personnel” and “function setting to meet the medical needs of community children” are the most important influencing factors; the weight of “urban and social integration” of criterion level is the smallest, 6.59%; 2. Among the overall community factors, the index of “building economized land” has the smallest weight, which is 0.29%. 3. Among the children’s factors, “safety and security” of the criterion layer has the largest weight, reaching 33.51%, and the “gaming and activity place” of the criterion layer has the smallest weight.

**Conclusions:** The building impact model of children’s community clinic with weight assignment can distinguish the important factors and the secondary factors in 68 specific indexes, which can provide the decision-making basis for the whole process of early planning, medium-term guidance and post-use evaluation, and realize the goal and effect of building completion.

**Acknowledgments**

1. Hunan Provincial Key Area R&D Program - Key R&D (2021WK2004), Foreign Expert Program of the Ministry of State Commission of Science Technology of China (DL2021160001L). 2. General Program of NSFC, 51978250, An Empirical Study on the Protection and Development of Traditional Villages in Minority Areas and Its Modern Spatial Transformation. 3. Natural Science Foundation of Hunan Province: 2022JJ30605, Hunan Provincial Department of Education Scientific Research Project: 21A0205.

## BEPS23027: PSYCHOLOGICAL ANALYSIS OF EMPLOYMENT STRESS OF COLLEGE STUDENTS AND THE ADJUSTMENT OF THE PROFESSIONAL TRAINING SYSTEM UNDER OBE MODE

Tan Ding^a^, Yun Lu^a^

^*a*^ *Wuchang Shouyi University, Wuhan, China.*

**Background:** This article aims to study the impact of employment pressure on the psychological health of college students who major in engineering by analyzing the differences in their exam scores of courses on Outcome based education (OBE) mode during different year. Based on the analysis proposed above, an adjustment method of courses on OBE mode for graduating students is proposed to reduce the psychological problems caused by employment pressure.

**Subjects and Methods:** This study selects 65 undergraduate students majoring in engineering as participants of the research subjects. Five exam scores of courses on OBE mode of each undergraduate are selected and compared with that of others. The comparation results show objective achievements of application-oriented courses on OBE mode are better than that of theoretical courses. At the same time, considering the psychological impact of employment pressure on college students during their graduation years, an adjustment method that can solve the above problems is proposed.

**Results:** The courses of the 65 college students are selected all belong to the course on OBE mode, and each course has 2 or 3 objective achievements except for total score. These objective achievements measure students’ mastery of course knowledge from different perspectives. In short, all objective achievements can be divided into three types, namely mastery of major theories or technologies, general application ability, and deep application ability. The scores and objective achievements of each course selected are compared with each other, and the comparation results show that the objective achievement of the theoretical comprehensive courses in their fourth year are the worst excluding the exam score of a course in the third year, while the objective achievement for another application-oriented course in their senior year is the best. From the perspective of the difficulty level of course knowledge, the selected senior courses are less difficult than other courses in sophomore and junior year. This indicates that learning goals of these students have already changed, and the change results from selective psychology under employment pressure.

**Conclusions:** This study selects five courses from 65 college students as research samples, which are all major courses on OBE mode in different years, including one course in the second year, two courses in the third year, and two courses in the fourth year. Comparing the objective achievements of each of the five courses selected with each other, we can find that the objective achievements of theoretical comprehensive courses in the fourth year are nearly the worst, while the objective achievements for another application course in the fourth year is the best. In addition, from the perspective of the difficulty and the role of the course in the talent training plan, the difficulty and importance of the course in the sophomore year are higher than that of other courses. From the perspective of the comprehensive level of knowledge structure, the theoretical comprehensive course in the fourth year has the highest comprehensive level. The reason for this is that students in their sophomore year are affected less by employment pressure and are able to learn important major courses attentively. However, in their senior year, employment pressure prompts students to select the key courses they think from all courses they are studying. All courses that can directly have a positive impact on all kinds of employment, including indirect employment or even implicit employment, are popular with graduating students. Therefore, the psychology of college students is greatly influenced by employment pressure in their fourth year. In order to ensure the education quality of students and their mental health, major establishment should be adapted to enable students to learn major knowledge well and find jobs easily. The adjustment methods include the following aspects: first all, adjusting the disciplinary training plan, bringing theoretical courses as early as possible, and adding practical training and employment skills training courses in the last year of undergraduate students; Then, administrators of universities should actively cooperate with enterprises, adjust teaching models based on theoretical teaching and guided by employment needs; finally, psychotherapy and guidance should be incorporated into the teaching process, and actively guide students to engage in psychological self-regulation, Enhance their psychological self-healing ability. The three measures of adjustment methods should be constructed from three aspects: external environment, self-awareness, and healing methods, which need to be tested in practice.

## BEPS23028: CONSTRUCTION AND PRACTICE OF AI ESCORT SYSTEM IN INTELLIGENT CLINIC

Juan Li ^a^

^*a*^ *The First Affiliated Hospital of University of Science and Technology of China, Hefei, Anhui 230001, China.*

**Background:** The Chinese government invests a large amount of funds in the medical and health field every year. As the main body of the medical service system, public hospitals play an important role in improving the fairness and accessibility of medical services. High-quality development and operation of hospitals cannot be separated from the support of information technology. In order to strengthen the construction of intelligent outpatient service and improve the operation efficiency of outpatient service, A grade-A3 hospital in eastern China launched an AI escort system in November 2022. Through the construction and practice of AI escort system, improve the efficiency of outpatient operation and management.

**Subjects and Methods:** A number of artificial intelligence technologies such as speech recognition, speech synthesis, semantic understanding, and virtual image are used to provide multi-modal AI interaction to guide and remind the whole process of medical treatment. Scientific research methods such as literature analysis, descriptive research and quantitative analysis were adopted to conduct statistical analysis on the functional application of AI diagnostic system, as well as the data of user usage and product interaction frequency distribution since the system was launched.

**Results:** The number of login users and the total number of interactions of the AI escort system were increasing continuously. The nodes that patients paid the most attention to in the process of medical treatment were registration success, registration, inspection and inspection appointment, inspection and inspection report, inspection and inspection completed, waiting status, etc. The online AI escort system shortens the waiting time of patients, improves the satisfaction of patients, and improves the order of outpatient treatment.

**Conclusions:** The AI escort system combines artificial intelligence technology with escort service, integrates AI technology with medical service through intelligent outpatient service, uses natural speech processing, machine learning and computer vision and other technologies to help patients improve the efficiency of treatment and improve the technical level of hospitals. Through AI technology, we can help patients achieve integrated management before, during and after diagnosis, provide patients with a higher level of medical service quality, and help hospitals develop in high quality. The AI escort system can also reduce the work burden of hospital staff and improve the allocation efficiency of medical resources.

## BEPS23029: ANALYSIS ON THE IMPACT OF EXERCISE THERAPY COMBINATION WITH DIETARY NUTRITION OF ELDERLY DIABETES NEGATIVE EMOTIONS AND ADHERENCE

Ming Wang^a^, Zehui Jiang^a^

^*a*^ *Zhuhai College of Science and Technology, Zhuhai, Guangdong 519041, China.*

**Background:** To observe and analyze the movement therapy in combination with dietary nutrition of elderly patients with diabetes mellitus (DM) negative emotions and compliance and therapeutic effect.

**Subjects and Methods:** From October 2020 to June 2022 of our hospital between 72 cases of elderly patients with diabetes were randomly divided into observation group and control group, each group 36 cases, including two groups were given conventional treatment, observation group while giving movement therapy in combination with dietary nutrition. Observe two groups of patients with negative emotions and compliance, and two groups HbAlc and blood lipid test results, the indicators and statistical analysis.

**Results:** Exercise therapy in combination with dietary nutrition observation group of patients after fasting blood glucose and postprandial 2 h blood glucose were significantly better than that of control group (P < 0.05). Treatment adherence observation group was 100%, the control group was 72.22% (26/36), the observation group of patients’ satisfaction was 100%, the control group was 83.33% (30/36), two groups of patients with negative emotions after exercise therapy in combination with dietary nutrition intervention has obviously improved, and the improvement of observation group was obviously higher than that of control group, P < 0.05, significant difference has statistical significance.

**Conclusions:** Exercise therapy in combination with dietary nutrition can improve the treatment compliance of elderly diabetes mellitus, and can improve the treatment effect of patients, worthy of clinical use.

**Acknowledgments**

Supported by “Three Levels” talent Construction Project of Zhuhai College of Science and Technology.

## BEPS23030: THE IMPACT OF DIGITAL TRANSFORMATION ON FACULTY WORKLOAD AND PSYCHOLOGICAL WELL-BEING IN HIGHER EDUCATION

Xiaoyan Qiao ^a,b^

^*a*^ *Department of Basic Education, Chongqing Hailian Vocational Technical College, Chongqing 400000, China,* ^*b*^ *Faculty of Business, Information & Human Science, Infrastructure University of Kuala Lumpur 43000, Kuala Lumpur, Malaysia*

**Background:** This study aims to explore the impact of digital transformation on the workload and psychological well-being of higher education professionals. As digital tools become increasingly integrated into education, understanding how this transformation affects the professional lives and psychological health of educators is crucial.

**Subjects and Methods:** This study employs a mixed-methods approach, combining qualitative interviews and structured questionnaires. In a 30-day online survey, 342 participants from diverse academic disciplines in higher education completed the questionnaire. Out of these, 300 valid responses were collected, accounting for 87.7% of the total. Qualitative interviews provided insights into the effects of digital transformation on the psychological well-being of educators.

**Results:** The findings reveal that a significant proportion of educators have incorporated digital tools into their teaching methods and vary in their comfort levels with technology use. Most participants perceive their task distribution as balanced or moderately balanced since the onset of digital transformation. While many did not perceive a significant impact of digital tools on their workload, a considerable portion acknowledged an increase in work-related stress due to the demands of digital transformation, which subsequently affects psychological well-being. Additionally, there are notable variations in job satisfaction among participants, with a substantial proportion expressing satisfaction or high satisfaction.

**Conclusions:** The study results indicate a positive correlation between the extent of digital transformation and workload perception, suggesting that as the influence of digital tools on workload increases, workload perception may also slightly increase, potentially affecting psychological well-being. Furthermore, a significant correlation exists between the stress induced by digital transformation and psychological well-being, highlighting the association between increased stress due to digital transformation demands and decreased psychological well-being. Moreover, a strong correlation between workload perception and psychological well-being is observed, implying that an increased workload perception may impact the psychological well-being of educators. These findings underscore the need to address workload and psychological well-being concerns of educators in the context of digital transformation and provide insights for higher education institutions to enhance their work environment.

## BEPS23031: AN IN-DEPTH STUDY ON THE EDUCATIONAL REFORM OF THE COURSE “MEDICAL DEVICE DEVELOPMENT AND PROMOTION”BASED ON THE PREMISE OF ABILITY CULTIVATION UNDER THE DQP FRAMEWORK IN THE UNITED STATES

Guifa Yao^a^, Wei Zhong^a^, Maorong Gu^b^, Yadi Chen^c^, Weiming Ye^d^, Huifang Chen^a,e,f^, Shuzhu Tian^a^

^*a*^ *Guangdong Lingnan Institute of Technology, Guangzhou, 510663, China,* ^*b*^ *Qingyuan Technician College, Qingyuan, 511515, China,* ^*c*^ *The University of New South Wales, Sydney, NSW 2052, Australia,* ^*d*^ *Zhongshan Shiqi Suhuazan Hospital, Zhongshan, 528402, China,* ^*e*^ *Guangxi Normal University, Guilin, 541004, China,* ^*f*^ *Guangzhou Huali College, Guangzhou, 511325, China.*

*GY and YC contributed equally to this study.*

**Background:** With the rapid development of China’s economy, the pharmaceutical industry is also rapidly rising. China’s higher pharmaceutical vocational education is also on the path of rapid development, undergoing rapid changes. The education models of nursing and pharmaceutical majors have also undergone significant changes. In order to implement the spirit of the Opinions on Implementing the National Demonstration Higher Vocational College Construction Plan and Accelerating the Reform and Development of Higher Vocational Education (JG [2006] No. 14), according to the the Pearl River Delta Regional Reform and Development Planning Outline (2008-2020) and the demand for talents in the medical device industry, and in line with the industrial development strategy of Lingnan Group, our hospital is conscious, planned Focus on building a “one body, two wings” disciplinary and professional system. Integrated “refers to the professional and professional groups supporting the development of the” Lingnan Health Industry “, including rehabilitation nursing, health management, medicine, health management, pharmaceutical technology, food and drug management, and other professional and professional groups. It is the main body of the “One Body, Two Wings”. The major of “Medical Device Maintenance and Management” is a key major in school construction, and “Medical Device Development and Promotion” is a key course of this major.

**Subjects and Methods:** The current traditional teaching models and curriculum systems have many problems, which can no longer meet the development needs of students, How to reform the education model and education system in the context of informatization has been the focus and difficulty of vocational education reform in recent years. In order to cultivate high-level talents in the field of “Medical Device Maintenance and Management”, we have carried out in-depth reforms on the series of courses in this field, especially the key course “Medical Device Development and Promotion”, which is based on the teaching reform of cultivating students’ abilities under the DQP (Degree Qualifications Profile) degree system in the United States.

**Results:** This article combines the current internationally advanced talent cultivation system for higher vocational education-the DQP (Degree Qualifications Profile) framework in the United States, which requires the ability requirements of the course “Medical Device Development and Promotion” in higher vocational education.

**Conclusions:** Based on the selection of different teaching methods, means, and reasonable organization of teaching content integration, the teaching of “Medical Device Development and Promotion” belongs to a new teaching mode, Deeply develop the curriculum standards for “Medical Device Development and Promotion”, organize the preparation of the latest engineering and academic combination of “Medical Device Development and Promotion” textbooks that meet the requirements of the times, and adopt a series of teaching reforms centered on students to enhance their self-learning ability through flipped classroom teaching, effectively stimulating students’ interest in learning and achieving good teaching results. This article explores and shares the comparison.

**Acknowledgments**

This work was supported by Grants from the research and practice of the school-level teaching and research project of Guangdong Lingnan Institute of Technology based on the training of teaching talents in the professional skills contest of electromechanical industrial robots (Project No.: JC202221).

## BEPS23032: EXPLORATION OF THE EFFECT OF NURSING INTERVENTION ON THE QUALITY OF LIFE OF ELDERLY HYPERTENSIVE PATIENTS AND ITS CLINICAL EFFECT

Na Li ^a^

^*a*^ *Pharmacy Intravenous Admixture Services, The Second Affiliated Hospital of Xingtai Medical College, Xingtai, Hebei, 054000, China.*

**Background:** Nursing intervention is a series of nursing activities based on a certain scientific theory and under the guidance of nursing diagnosis according to a predetermined intervention method. Nurses determine nursing interventions according to the characteristics of nursing diagnosis, nursing research results, the potential of patients’ functional rehabilitation, and the abilities of patients and nurses themselves. Interventions help patients achieve their intended goals: prevent complications and promote, maintain, or restore physical and mental function. The prevalence of hypertension in the elderly population in China is as high as 49%. In the early days, people believed that hypertension in the elderly was a physiological phenomenon that blood pressure increased with age, and there was no need to treat it. However, long-term studies have shown that hypertension in the elderly is an important factor endangering the survival and quality of life of the elderly, and active treatment can significantly reduce the risk of important cardiovascular events such as stroke. Regardless of age, blood pressure should be controlled under the guidance of a doctor to reduce it as far as possible to the normal range. This article evaluated the effect of nursing intervention on the quality of life of elderly patients with hypertension and the effect of clinical intervention.

**Subjects and Methods:** 100 cases of elderly hypertensive patients were selected from December 2021 to November 2022 as the subjects of this study, and the nursing intervention was carried out for a period of 5 months through the establishment of a health clinical file card, the adoption of psychological guidance, health education, exercise and behavioral guidance, dietary control, scientific medication, etc. The patients’ hypertensive conditions were also compared before and after the intervention.

**Results:** After the nursing intervention, the patients’ hypertension awareness rate, medication taking rate, blood pressure control rate, and the score of the comprehensive quality of life questionnaire were significantly improved, and these changes were statistically significant. The psychological, physical, social, and material well-being of elderly hypertensive patients have significantly improved, especially in terms of physical function, psychological, and material well-being.

**Conclusions:** Therefore, the implementation of effective nursing intervention for elderly patients with hypertension is an effective way to maintain stable blood pressure, reduce complications, improve patients’ self-management ability and quality of life, and has the value of comprehensive promotion. Through systematic nursing intervention and effective health education, patients’ cognition of hypertension can be improved, so that they can have a more comprehensive understanding of the disease, including diagnostic criteria, risk factors and complications, so as to enhance self-care awareness, improve the quality of life of residents, and reduce the social burden.

## BEPS23033: EFFECT OF EARLY INTERVENTION OF MENTAL HEALTH EDUCATION ON THE DEGREE OF DEPRESSION OF COLLEGE LIBRARIAN

Lihong Duan ^a^

^*a*^ *Wuchang Shouyi University, 430074, Wuhan, Hubei, China.*

**Background:** This study aims to investigate the effect of early intervention of mental health education on the degree of depression of college librarian.

**Subjects and Methods:** This study focuses on university librarians. This survey randomly selected 600 librarians from various universities who have worked for 1-4 years. Based on librarians with different years of service, combined with the characteristics and specificity of the questionnaire, this study used the UPI personality questionnaire for new librarians who have only worked for one year. The SCL-90 Symptom Checklist is used for librarians who have been working for 2-4 years.

**Results:** The average score of SCL-90 factors in mental health of university librarians is higher than the national norm. There were significant differences in the average scores of depression, anxiety, paranoia, and psychiatric factors. From a gender perspective, the mental health status of female university librarians is not as good as that of male librarians. There are significant differences in 9 factors among university librarians with different working years. Overall, there is a downward trend as the number of years worked increases.

**Conclusions:** (1) Improving awareness, update concepts, and strengthen mental health education; (2) Constructing a highly operable mental health education system; (3) Combining mental health education with moral education to improve work efficiency; (4) Strengthen the construction of campus culture and create a good campus cultural environment; (5) Improving the mental health of librarians.

## BEPS23034: REGULATORY EFFECT OF ROSENTHAL EFFECT ON STUDENTS’ PSYCHOLOGICAL QUALITY AND EMOTIONAL STABILITY UNDER OBE MODE

Yun Lu^a^, Xinxing Wu^b^, Xiaofeng Li^c^

^*a*^ *School of Mechatronics and Automation, Wuchang Shouyi University, Wuhan, Hubei 430064, China,* ^*b*^ *Wuhan University of Technology, Wuhan, Hubei 430070, China,* ^*c*^ *Department of Public Teaching, Dalian University of Finance and Economics, Dalian 116622, China.*

**Background:** This study aims to investigate the effect of Rosenthal effect on students’ psychological quality and emotional stability under OBE mode.

**Subjects and Methods:** This study focuses on university students. Before practical teaching, 61 students were evaluated using the SCL-90 scale. 25 students with significantly higher scores than the norm were assigned to the anxiety group. Introducing the Rosenthal effect teaching method into the practical teaching and comprehensive training project assessment of “Engineering Testing and Signal Processing” in the anxiety group. 36 nursing students with no statistically significant difference in scores compared to the norm were assigned to the non anxiety group and traditional teaching methods were used.

**Results:** Comparison of somatization, compulsion, anxiety, and interpersonal sensitivity in SCL-90 between two groups before practical teaching and two comprehensive project training assessments showed that the main effects of intervention were all *p*<0.05. The performance of the anxiety group in two practical training assessments was significantly better than that of the non anxiety group (both *p*<0.05). The comparison of emotional stability scores in each group’s examination was F=0.89l, *p*=0.412. After stratification according to the degree of focus, the comparison results showed that F=2.667, *p*=0.017, and the pairwise comparison showed that there were statistical differences between the ultra-high focus group and the high focus group compared to the low focus group, medium low focus group, and control group (*p*<0.05). The former is lower than the latter. Comparison of trait anxiety scores between two groups: F=0.163, *p*=0.850. The results of comparing the state anxiety scores of each group of testers before and after the examination using a repeated measurement design analysis of variance were: F=53.704 before and after the examination, *p*=0.000. F=1.246 and *p*=0.290 between groups. The interaction between the front, back, and between groups F=0.239, *p*=0.780. The adjusted mean comparison of state anxiety scores among each group after examination F=1.546, *p*=0.216. The results of comparing the systolic blood pressure of each group before, during, and after the examination using a repeated measurement design analysis of variance showed that F=5.564 before, during, and after the examination, *p*=0.004. F=0.609 and *p*=0.545 between groups. The interaction between the front, middle, and back groups F=0.820, *p*=0.513. Before, during, and after the examination, there was a statistically significant difference (*p*=0.002) between the two groups, with the former being lower than the latter. The adjusted mean of systolic blood pressure in each group was compared with F=1.116 and *p*=0.330 during the examination; After inspection, compare F=0.039 and *p*=0.962. The results of comparing the diastolic blood pressure before, during, and after each group examination using a repeated measurement design analysis of variance showed that F=19.166, *p*=0.000, and F=1.137, *p*=0.323 between groups. The interaction between the front, middle, and back groups F=0.416, *p*=0.797. Before, during, and after the examination, there was a statistical difference (*p*=0.000) between the two groups, with the former being lower than the latter. The adjusted mean of diastolic blood pressure in each group was compared with F=0.478 and *p*=0.621 during the examination. After inspection, compare F=0.855 and *p*=0.421. The results of comparing the heart rates before, during, and after each group’s examination using repeated measurement design analysis of variance. Before, during, and after inspection, F=26.013, *p*=0.000. F=0.905 and *p*=0.407 between groups. The interaction between the front, middle, and back groups F=0.595, *p*=0.650. Before, during, and after the examination, there was a statistical difference (*p*=0.000) between the two groups, and the results were faster in the front, middle, and back. The adjusted mean of heart rate in each group was compared with F=0.490 and *p*=0.613 during the examination; After inspection, compare F=0.386 and *p*=0.680. Comparison of willingness to undergo re-examination rates among three groups χ^2^=6.632, *p*=0.036. There was a statistical difference (*p*<0.05) between the pairwise comparison and the control group.

**Conclusions:** The Rosenthal effect can enhance students’ psychological quality and alleviate their anxiety about experimental skills and operations. The Rosenthal effect can improve students’ classroom attention and experimental skill performance.

**Acknowledgement**

This paper is supported by Doctoral Research Start-Up Fund of Wuchang Shouyi University in 2022 and Undergraduate First-class Courses Fund of Wuchang Shouyi University with Grant No. 2023XC01.

## BEPS23035: EXPLORING THE CORRELATION BETWEEN EPIDEMIC DENSITY AND COMMUNITY SPATIAL DENSITY IN HIGH-DENSITY URBAN AREAS FROM THE PERSPECTIVE OF PUBLIC HEALTH: A CASE STUDY OF HUANGPU DISTRICT, SHANGHAI

Jian Li^a^, Yu Shi^a^, Qingchang Chen^a,b^

^*a*^ *School of Urban Construction and Safety Engineering, Shanghai Institute of Technology, Shanghai 201418, China,* ^*b*^ *Shanghai Style Architecture Culture Inheritance and Innovation Center, Shanghai, China.*

**Background:** In early March 2022, a major outbreak of infectious disease occurred in Shanghai, leading to the implementation of global static management measures in the city. This had a profound impact on the daily lives of residents as well as social and economic development. The rapid transmission of the virus not only posed significant threats to physical health but also induced panic and adversely affected mental well-being. Consequently, addressing public health emergencies in densely populated urban areas has emerged as a crucial research focus within disciplines such as urban planning and medicine.

**Subjects and Methods:** Based on the background of public health emergencies, this study explores the relationship between high-density urban areas and public health from the perspective of infectious diseases. This paper analyzes the epidemic density in Shanghai during the outbreak period using published case data, and subsequently establishes a regression model of epidemic density and physical spatial density with Huangpu District as a representative case. The principal component analysis was employed to extract four principal components that characterize the layout and planning attributes of the residential area. The method of geographical weighted regression was utilized to examine both the direction and magnitude of their impact on the spatial distribution patterns observed in relation to epidemics.

**Results:** High-density urban areas offer enhanced infrastructure, yet also entail alterations in living conditions and lifestyles. Urban inhabitants encounter a range of health challenges, including climate and environmental hazards, chronic illnesses, physical inactivity, and mental health issues that are significantly impacted by public health emergencies. The findings indicate a positive correlation between the scale of community construction and epidemic density, while epidemic density was negatively associated with community fold degree and environmental quality. Moreover, the accessibility of community service facilities demonstrated both positive and negative associations with epidemic density.

**Conclusions:** The findings indicate that the spatial-temporal diffusion of the epidemic can be managed by mitigating residential density under specific circumstances. It is crucial to expedite the renovation of outdated residential areas in densely populated urban regions, enhance the quality of residential environmental hygiene, rationally arrange high-rise buildings, and improve public service facilities in living quarters. The urban planning departments and health and epidemic prevention departments should collaborate in the future to establish a comprehensive health and epidemic prevention system at various scales within cities, communities, and buildings. Furthermore, the utilization of high-density urban areas’ infrastructure and resources to enhance public health is a crucial concern in both planning and medical fields.

**Acknowledgments**

This work was supported by a project grant from 2023 Shanghai Education Science Research Project: Research on Practical Teaching Evaluation System of Applied Undergraduate Digital Scene Design under the Urban Metaverse (C2023104).

## BEPS23036: ANXIETY ALLEVIATION AND EMOTIONAL REGULATION EFFECTS OF EMOTIONAL THERAPY ON UNIVERSITY JOURNAL EDITORS

Xinxing Wu^a^, Yun Lu^b^, Xiaofeng Li^c^

^*a*^ *Wuhan University of Technology, Wuhan, Hubei 430070, China,* ^*b*^ *School of Mechatronics and Automation, Wuchang Shouyi University, Wuhan, Hubei 430064, China,* ^*c*^ *Department of Public Teaching, Dalian University of Finance and Economics, Dalian 116622, China.*

**Background:** This study aims to investigate the anxiety alleviation and emotional regulation effects of emotional therapy on university journal editors.

**Subjects and Methods:** 100 university journal editors are selected and randomly divided into two groups, each with 50 cases. The control group received commonly used psychological interventions. The observation group adopted emotional therapy on the basis of commonly used psychological interventions. Evaluate the coping styles, negative emotions, and stress tests of two groups of university journal editors.

**Results:** The positive coping style scores of two groups of university journal editors increased compared to before intervention, and the increase was more significant in the observation group. The negative coping style score decreased, and the observation group showed a more significant decrease. The differences were statistically significant (*p*<0.05). The depression and anxiety scores of the two groups of university journal editors decreased compared to before the intervention. After the intervention, the depression and anxiety scores of the observation group were significantly lower than those of the control group. The stress score of the observation group’s family members after intervention was significantly lower than that of the control group. The differences were statistically significant (*p*<0.05). Emotions, as an important aspect of editors’ psychological life, to a certain extent affect their work, mood, and passion. The goal of emotional regulation in editors is to unleash their positive emotions, overcome negative emotions, and minimize their emotional states of passion and stress.

Stress is the three states of emotions. Different emotional states have different impacts on editing work.

**Conclusions:** The application of emotional therapy in anxiety relief and emotional regulation of university journal editors can help stabilize their emotions. This can encourage them to choose reasonable coping methods, actively cooperate with psychological workers, and reduce their internal pressure.

**Acknowledgement**

This paper is supported by Doctoral Research Start-Up Fund of Wuchang Shouyi University in 2022 and Undergraduate First-class Courses Fund of Wuchang Shouyi University with Grant No. 2023XC01.

## BEPS23037: A NETWORK PHARMACOLOGY APPROACH TO INVESTIGATE THE UNDERLYING MECHANISMS OF ALPINIA KATSUMADAI HAYATA ON ACNE VULGARIS

Meiyan Fu^a^, Shanshan Huang^b^, Huifang Chen^b,c,d^, Dongyong Luo^e^, Jun Sun^f^, Xiaofeng Wu^b^, Hong Xia^g^, Jianhua Yi^h^

^*a*^ *Guangzhou Phgo Biotechnology Co., Ltd., Guangzhou, 510507, China,* ^*b*^ *Guangdong Lingnan Institute of Technology, Guangzhou, 511486, China,* ^*c*^ *Guangxi Normal University, Guilin, 541004, China,* ^*d*^ *Guangzhou Huali college, Guangzhou, 511325, China,* ^*e*^ *People's Hospital of Shenzhen Guangming District, Shenzhen, 518107, China,* ^*f*^ *Guangzhou Institute of Advanced Technology, Chinese Academy of Sciences, Guangzhou 511458, China,* ^*g*^ *Guangzhou Panyu District Central Hospital, Guangzhou, 510663, China,* ^*h*^ *East Hospital of the First Affiliated Hospital of Sun Yat sen University, Guangzhou, 510700, China.*

*MF and HC contributed equally to this study.*

**Background:** The Alpinia katsumadai Hayata (Doukou, DK) is a traditional Chinese medicine with excellent anti-inflammatory properties, widely used in the food and commodity industries. Because cardamom has the effect of appetizing and digesting the spleen and stomach meridians, it can enhance the function of the spleen and stomach, facilitate food digestion, and improve symptoms such as lack of appetite, persistent food accumulation, chest and abdominal pain, and chest tightness and hunger. Cardamom has a warm nature and can also have a warming and anti nausea effect, relieving symptoms of cold, dampness, and nausea. In addition, cardamom has the effect of dissipating dampness and promoting qi circulation, improving the function of spleen and stomach circulation, and promoting normal water and grain circulation. Modern pharmacological studies have shown that cardamom has anti asthma and antibacterial effects. Cardamom is a common Chinese herbal medicine with various effects and functions, which can be used for regulating physical health, beauty and beauty, treating acne vulgaris, and other aspects. In terms of regulating gastrointestinal function: Cardamom can promote gastrointestinal peristalsis, alleviate gastrointestinal discomfort, and alleviate symptoms such as diarrhea. In terms of smell improvement: cardamom has a fresh fragrance, which can be used in oral fresheners, perfume, etc., as well as in cooking to improve the smell of dishes. In terms of antioxidant properties: Cardamom contains rich antioxidant substances, which can help eliminate free radicals, delay aging, and prevent diseases such as cancer. In terms of freckle removal, whitening, and treatment of acne vulgaris, the essential oil components in cardamom can promote skin blood circulation, fade black spots, and whiten the skin. In terms of regulating endocrine function: The components in cardamom can regulate female endocrine function, alleviate menstrual discomfort, and regulate menstruation. It should be noted that although cardamom has multiple functions and effects, it is necessary to pay attention to the dosage when using it, and do not consume or overuse cardamom products such as cardamom essential oil. Meanwhile, pregnant and lactating women should not use cardamom.

**Subjects and Methods:** A total A network pharmacology analysis was performed to identify the potential anti-acne compounds, hub therapeutic targets, and the key pathways via TCMSP, BATMAN, CTD, PDB and PubChem databases. Finally, the “compound-target-pathway” network was constructed.

**Results:** The study found total 7 active compounds, including quercetin, (2R)-5,7-dihydroxy-2-phenylchroman-4-one, dehydrodiisoeugenol, (2R)-7-hydroxy-5-methoxy-2-phenylchroman-4-one, Pinocembrin, and 1,7-diphenyl-5-hydroxy-6-hepten-3-one alpinolide peroxide. In addition, 30 therapeutic targets, and 4 hub therapeutic targets of the DK were identified. The biological processes were primarily related to inflammatory response, response to oxidative stress, regulation of insulin secretion, etc. Which was significantly associated with ten pathways including the PI3K-Akt signaling pathways, VEGF signaling pathways, etc. Furtherly, the 4 hub targets AKT1, F2, AR, and PTGS2 with higher connectivity in PPI network were verificated though molecular docking, which once again proved that these targets are potential targets of their corresponding chemical molecules.

**Conclusions:** DK might have a synergistic effect on the anti-inflammatory effects via the various active compositions, targets and signaling pathways.

**Acknowledgments**

This work was supported by grants from the Project No. 20171190 of Guangdong Administration of traditional Chinese Medicine

## BEPS23038: CLINICAL VALUE OF SERUM FABP4, CXCL12 AND INFLAMMATORY FACTORS IN PATIENTS WITH ACUTE EXACERBATION OF CHRONIC OBSTRUCTIVE PULMONARY DISEASE

Xuejie Zhai^a^, Ruling Dai^a^

^*a*^ *Department of Respiratory and Critical Care Medicine, Zhuanghe Central Hospital, Dalian 116400, Liaoning Province, China.*

**Background:** The study, titled “Clinical Value of Serum FABP4, CXCL12 and Inflammatory Factors in Patients with Acute Exacerbation of Chronic Obstructive Pulmonary Disease”, aims to investigate the potential role of fatty acid binding protein 4 (FABP4), inflammatory chemokine CXCL12, and various inflammatory factors in the acute exacerbation of chronic obstructive pulmonary disease (AECOPD). The study focuses on the correlation of these factors with AECOPD, their relationship with other inflammatory markers, and their utility in assessing the severity and prognosis of the disease. Chronic obstructive pulmonary disease (COPD) is a widespread and preventable illness that is often characterized by continuous respiratory symptoms and airflow constraints. These symptoms tend to progress over time and are generally linked to an intensified chronic inflammatory response in the airways and lungs, usually due to harmful particles or gases. Acute exacerbation of COPD (AECOPD), a critical event in the course of COPD, is the most common cause for medical consultation and mortality linked to the disease. Identifying biomarkers that can accurately reflect the inflammatory status and severity of AECOPD is vital for prognosis assessment and treatment guidance.

**Subjects and Methods:** M In this study, 62 patients diagnosed with AECOPD were selected for observation, and 40 healthy subjects undergoing physical examination in the same period were used as the control group. The levels of serum FABP4, CXCL12, interleukin-6 (IL-6), and C-reactive protein (CRP) were measured in both groups, and the pulmonary function and clinical data of the patients were collected for correlation analysis.

**Results:** The results of the study showed that the levels of FABP4, CXCL12, IL-6, and CRP were significantly higher in the observation group compared with the control group. FABP4 was positively correlated with CXCL12, and both FABP4 and CXCL12 were positively correlated with IL-6 and CRP. Interestingly, FABP4 and CXCL12 were negatively correlated with pulmonary function parameters. Furthermore, the levels of FABP4 and CXCL12 in the group that did not survive were significantly higher than those in the group that did survive.

**Conclusions:** From these results, the study concluded that FABP4 and CXCL12 play a crucial role in the development of AECOPD. They are related to the inflammatory response and pulmonary dysfunction and can be used as indicators for judging the severity of inflammation and poor prognosis of AECOPD. However, the study also acknowledges that the pathogenesis of AECOPD is complex and may involve various inflammatory pathways and signal transduction processes. The exact mechanisms of action for FABP4 and CXCL12 in the development of AECOPD require further investigation.

